# TET3 epigenetically controls feeding and stress response behaviors via AGRP neurons

**DOI:** 10.1172/JCI162365

**Published:** 2022-10-03

**Authors:** Di Xie, Bernardo Stutz, Feng Li, Fan Chen, Haining Lv, Matija Sestan-Pesa, Jonatas Catarino, Jianlei Gu, Hongyu Zhao, Christopher E. Stoddard, Gordon G. Carmichael, Marya Shanabrough, Hugh S. Taylor, Zhong-Wu Liu, Xiao-Bing Gao, Tamas L. Horvath, Yingqun Huang

**Affiliations:** 1 Department of Obstetrics, Gynecology and Reproductive Sciences,; 2 Yale Center for Molecular and Systems Metabolism, and; 3 Department of Comparative Medicine, Yale University School of Medicine, New Haven, Connecticut, USA.; 4 Department of Biostatistics, Yale School of Public Health, New Haven, Connecticut, USA.; 5 Department of Genetics and Genome Sciences, University of Connecticut Health Center, Farmington, Connecticut, USA.; 6 Department of Neuroscience, Yale University School of Medicine, New Haven, Connecticut, USA.

**Keywords:** Metabolism, Neuroscience, Diabetes, Leptin, Neuroendocrine regulation

## Abstract

The TET family of dioxygenases promote DNA demethylation by oxidizing 5-methylcytosine to 5-hydroxymethylcytosine (5hmC). Hypothalamic agouti-related peptide–expressing (AGRP-expressing) neurons play an essential role in driving feeding, while also modulating nonfeeding behaviors. Besides AGRP, these neurons produce neuropeptide Y (NPY) and the neurotransmitter GABA, which act in concert to stimulate food intake and decrease energy expenditure. Notably, AGRP, NPY, and GABA can also elicit anxiolytic effects. Here, we report that in adult mouse AGRP neurons, CRISPR-mediated genetic ablation of *Tet3*, not previously known to be involved in central control of appetite and metabolism, induced hyperphagia, obesity, and diabetes, in addition to a reduction of stress-like behaviors. TET3 deficiency activated AGRP neurons, simultaneously upregulated the expression of *Agrp*, *Npy*, and the vesicular GABA transporter *Slc32a1*, and impeded leptin signaling. In particular, we uncovered a dynamic association of TET3 with the *Agrp* promoter in response to leptin signaling, which induced 5hmC modification that was associated with a chromatin-modifying complex leading to transcription inhibition, and this regulation occurred in both the mouse models and human cells. Our results unmasked TET3 as a critical central regulator of appetite and energy metabolism and revealed its unexpected dual role in the control of feeding and other complex behaviors through AGRP neurons.

## Graphical abstract

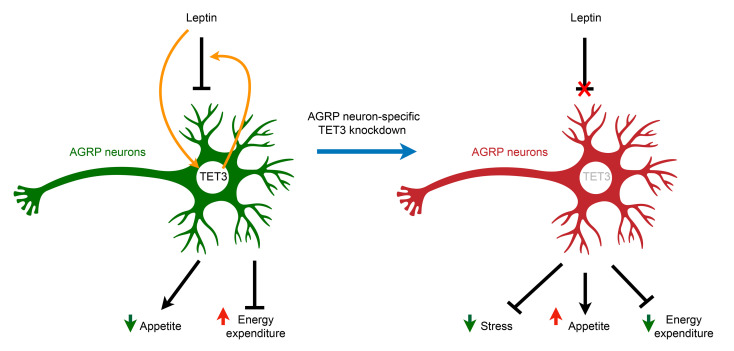

## Introduction

The capacity of the brain to balance between energy intake and expenditure is a critical aspect of survival. The agouti-related peptide–expressing (AGRP-expressing) neurons located in the hypothalamic arcuate nucleus (ARC) play an essential role in appetite control and energy homeostasis (1–3). Ablation of these neurons in adult mice leads to starvation and eventual death (4, 5). Energy deprivation activates AGRP neurons leading to increased production and release of AGRP, neuropeptide Y (NPY), and the neurotransmitter GABA, which act in concert to drive food intake and decrease energy expenditure (1–3). Notably, the vesicular GABA transporter (VGAT) encoded by the *Slc32a1* gene is required for vesicular accumulation of GABA, and its deletion results in a complete loss of synaptic GABA release (6, 7). Leptin is an adipocyte-derived peptide hormone that functions in the brain to suppress appetite, promote energy expenditure, and maintain glucose homeostasis (8–10). Dysregulation of leptin or its receptor (LEPR) causes severe obesity and diabetes (11–13). In AGRP neurons, leptin signaling inhibits the expression of both *Agrp* and *Npy* (14). Recently, AGRP neurons have been identified as the primary target of leptin action in a seminal study demonstrating that AGRP neuron–specific *Lepr* deletion at the adult stage in mice causes severe hyperphagia, obesity, and diabetes (15). Although initially hailed as a potential treatment for obesity and diabetes, the clinical applications of leptin have remained limited, owing at least in part to an incomplete understanding of the mechanism by which leptin regulates gene expression including that of key neuropeptides such as AGRP and NPY (16, 17). AGRP neurons have also been implicated in circuitry control of nonfeeding behaviors including those associated with reward, anxiety, compulsiveness, depression, and voluntary exercise (18–25). However, the molecular basis of the pleiotropic action of these neurons on multiple behaviors has remained elusive.

The TET family of dioxygenases (TET1/-2/-3) initiate DNA demethylation by converting 5-methylcytosines (5mC) to 5-hydroxymethylcytosines (5hmC), which they further oxidize into 5-formylcytosines (5fC) and 5-carboxylcytosines (5caC), which are removed by thymine DNA glycosylase, completing the cytosine demethylation cycle (26–28). 5hmC also serves as a stable epigenetic mark and functions to enhance or inhibit the binding of regulatory protein factors in a context-dependent manner (29, 30). TETs can also regulate chromatin architecture and gene transcription independently of their catalytic activities (31–37). In the mouse brain, *Tet* genes are widely transcribed across different forebrain regions including the cortex, hippocampus, cerebellum, and hypothalamus, with *Tet3* being the most abundant (38, 39). TET1 and TET2 have been shown to play important roles in learning and memory processes in adult mice (32, 40–43). While *Tet3* knockout in mice is neonatally lethal (44), *Tet3* knockdown in the infralimbic prefrontal cortex or hippocampal neurons impairs fear extinction memory (45, 46). In adult mice, *Tet3* ablation in forebrain neurons (particularly hippocampal neurons) results in increased anxiety and impaired spatial orientation (47). These studies have relied on non–cell type–specific approaches, and a clear mechanistic understanding linking cell type–specific epigenetic changes induced by TETs to specific behavioral phenotypes has not been accomplished. In addition, there is a paucity of information regarding the potential role of TETs in central control of energy metabolism, which is addressed in this study.

## Results

### TET inhibition elevates AGRP expression in the ARC.

We have previously shown that TET3 expression is aberrantly elevated in the livers of humans and mice with type 2 diabetes and that liver-specific, siRNA-mediated TET3 knockdown improves glucose homeostasis both in dietary and genetic mouse models of diabetes (48). Bobcat339 (Bobcat) is a synthetic cytosine derivative capable of binding competitively with 5mC to the active sites of TETs and inhibits their enzymatic activity in cultured neuronal cells (49). We hypothesized that Bobcat might have an antidiabetic effect owing to its ability to inhibit TETs. Thus, we treated high-fat diet–induced (HFD-induced) diabetic mice with Bobcat in the drinking water for 2 weeks. Unexpectedly, the Bobcat-treated animal became hyperphagic, leading to speculation that Bobcat might have affected the brain. Thus, we treated nondiabetic mice fed a regular chow with Bobcat in the drinking water. Four days later, ARCs were isolated from ad libitum–fed mice and analyzed. As shown in Supplemental Figure 1A, Bobcat treatment increased AGRP expression in the ARC, which at least in part explained why the mice became hyperphagic. In light of these observations, we began to explore TETs in AGRP neurons for a potential role in the control of feeding and energy metabolism.

### Food deprivation downregulates TET3 in AGRP neurons.

**Figure 1:**
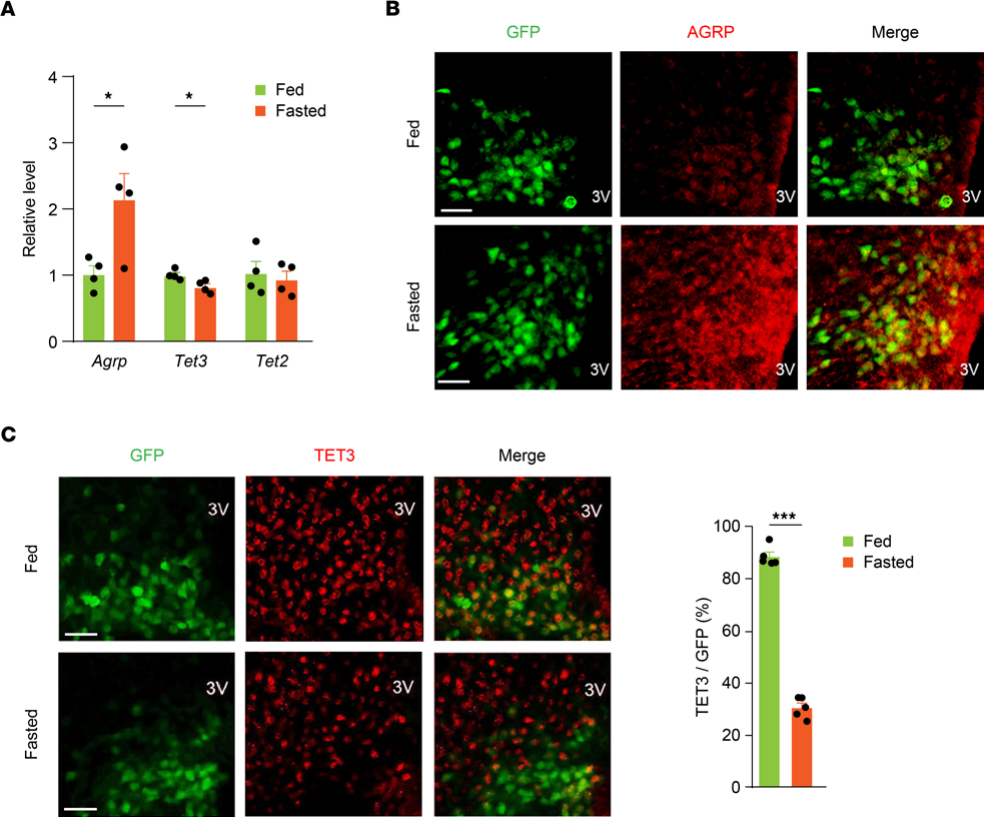
Fasting decreases TET3 expression in AGRP neurons. (**A**) Levels of *Agrp*, *Tet3*, and *Tet2* mRNA in the ARCs of fed and fasted mice. *n =* 4 mice per group. *P < 0.05, by 2-tailed Student’s *t* test. (**B**) Representative photomicrographs of AGRP neurons (green) and AGRP (red) showing that AGRP expression markedly increased in the ARCs of fasted mice. Scale bars: 50 μm. (**C**) Representative photomicrographs and corresponding statistical analysis of TET3^+^ (red) AGRP neurons (green) showing decreased TET3 expression in AGRP neurons in fasted mice. *n =* 5 mice per group. ***P < 0.001, by 2-tailed Student’s *t* test. 3V, third ventricle. Scale bars: 50 μm. All data represent the mean ± SEM.

To determine whether fasting, which normally upregulates AGRP, would affect TET expression in AGRP neurons, we mated *Agrp-IRES-Cre* mice with Cre-enabled *Rosa26-LSL-Cas9-GFP*–knockin mice to obtain *Agrp-IRES-Cre:LSL-Cas9-GFP* mice (hereafter called Cas9^+^ mice), which coexpress GFP and Cas9 endonuclease specifically in AGRP neurons. The Cas9^+^ mice were fasted overnight for 12 hours, and ARCs were isolated for RNA and protein analyses. Fasting increased *Agrp* mRNA by approximately 2-fold as compared with ad libitum–fed mice (Figure 1A). Previous cell type–specific transcriptome sequencing using purified mouse AGRP neurons showed an approximately 4-fold increase in *Agrp* mRNA following a 24-hour fast (50). Fasting decreased *Tet3* mRNA expression without affecting *Tet2* mRNA in the ARC (Figure 1A). *Tet1* expression in the ARC was negligible (data not shown). Immunofluorescence analysis revealed a marked increase in AGRP in the ARC of fasted versus fed mice (Figure 1B). The diffuse, robust AGRP signal was consistent with AGRP being a secreted, stable peptide and AGRP neurons’ broad projections into other areas of the brain (3, 51). Although TET3 protein was readily detected in both AGRP and non-AGRP cells, fasting clearly decreased the number of TET3^+^ AGRP neurons (Figure 1C). The apparently modest decrease in *Tet3* mRNA expression by fasting (Figure 1A) versus protein expression (Figure 1C) was likely, in part, a result of using a mixed cell population of the ARC in the reverse transcription–quantitative PCR (RT-qPCR) assays. The specificity of the TET3 antibody was previously validated (48) and further confirmed using an siRNA specifically targeting mouse *Tet3* (*Tet3* siRNA) in a mouse hypothalamic neuronal cell line (Supplemental Figure 1; supplemental material available online with this article; https://doi.org/10.1172/JCI162365DS1). Taken together, our results demonstrated that fasting downregulated *Tet3* expression in AGRP neurons.

### TET3 knockdown upregulates AGRP in AGRP neurons.

**Figure 2:**
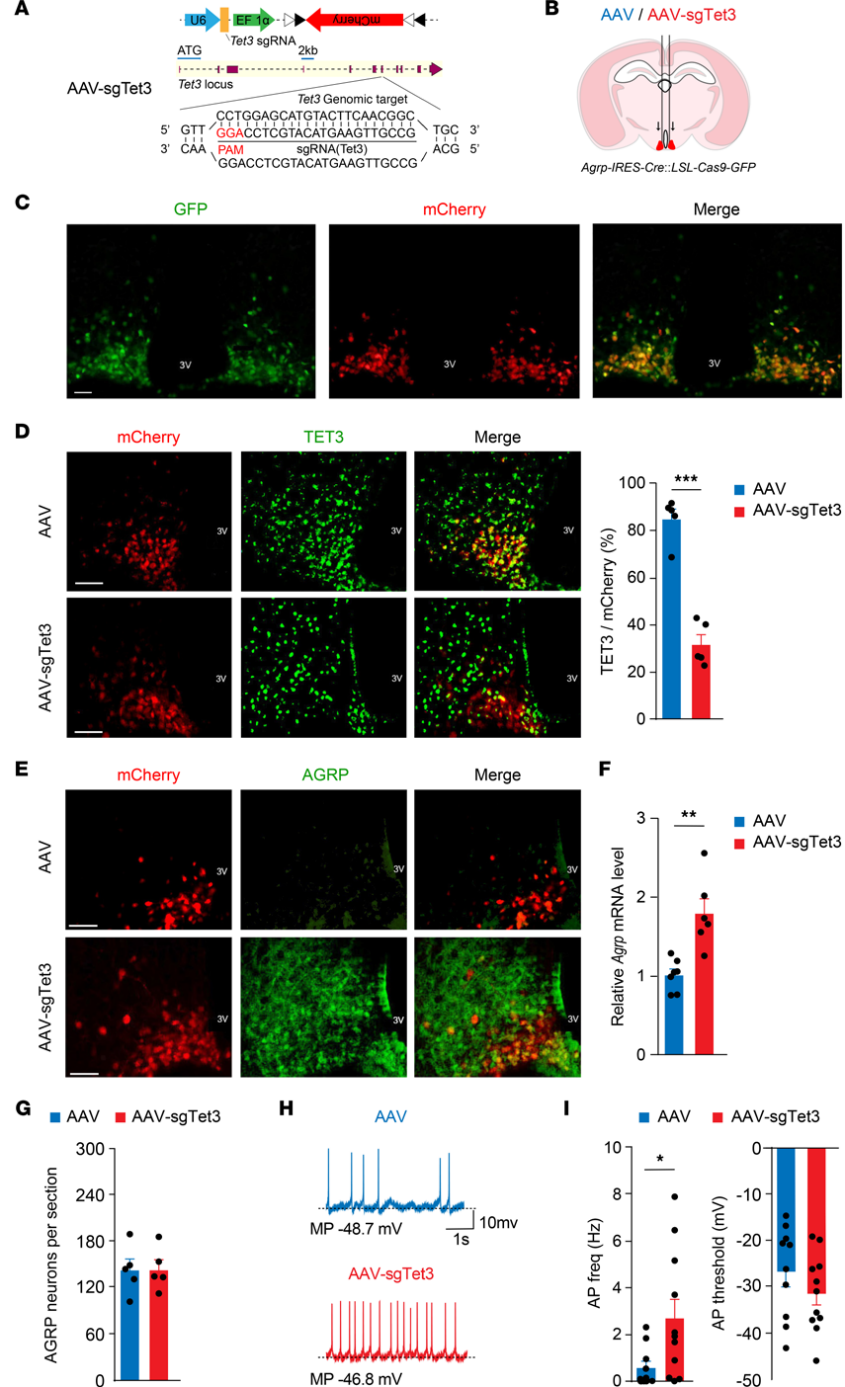
TET3 knockdown increases AGRP expression and AGRP neuronal activity. (**A**) Schematic diagram of AAV-sgTet3 (top) with the sgRNA design for targeting the mouse *Tet3* genomic locus (bottom). (**B**) Schematic diagram of bilateral virus injection into the ARC of Cas9^+^ mice. (**C**) Representative photomicrographs of AGRP neurons (green) expressing injected AAV-sgTet3 (red). (**D**) Representative photomicrographs and corresponding statistical analysis of TET3^+^ (green) AGRP neurons (red) showing decreased TET3 expression in AGRP neurons in AAV-sgTet3–injected mice. *n* = 5 mice per group. ***P < 0.001, by 2-tailed Student’s *t* test. (**E**) Representative photomicrographs of AGRP neurons (red) and AGRP (green) showing a marked increase in AGRP in the ARC of ad libitum–fed mice injected with AAV-sgTet3. (**F**) Increased expression of *Agrp* mRNA in the ARC of mice injected with AAV-sgTet3. *n* = 6–7 mice per group. **P < 0.01, by 2-tailed Student’s *t* test. (**G**) Quantification of AGRP neurons in the ARCs of Cas9^+^ mice injected with AAV or AAV-sgTet3 showing no significant difference between the groups. *n =* 5 mice per group. Significance was determined by 2-tailed Student’s *t* test. (**H**) Representative traces of membrane and APs recorded under current clamp in AGRP neurons of Cas9^+^ mice injected with AAV or AAV-sgTet3. (**I**) Bar graphs show the frequency of spontaneous APs (AP freq, left) and AP threshold (right) in AGRP cells in control and TET3-knockdown mice. *n* = 10–11 neurons from 3–4 mice per group. *P < 0.05, by 2-tailed Student’s *t* test. All data represent the mean ± SEM. Scale bars: 50 μm (**C**–**E**).

To directly evaluate the functional significance of TET3 in AGRP neurons, we used CRISPR gene-editing technology to downregulate TET3 specifically in AGRP neurons. We created an adeno-associated virus (AAV) vector containing a single-guide RNA targeting the mouse *Tet3* locus (sgTet3) and a Cre-dependent mCherry reporter to indicate virus-transduced neurons (AAV-sgTet3, Figure 2A). The sgTet3 sequence has been extensively validated for lack of CRISPR-mediated off-target mutagenesis (52). AAV-sgTet3 or negative control AAVs (53) was injected bilaterally into the ARC of Cas9^+^ mice (Figure 2B), and AGRP neuron–specific expression of sgTet3 was confirmed by the presence of GFP and mCherry double-positive cells (Figure 2C). To examine the effects of TET3 knockdown in AGRP neurons, ARCs were isolated (9:00 am–10:00 am) from fed mice injected with AAV-sgTet3 or AAV viruses. While the protein signal of TET3 in AAV-sgTet3–transduced AGRP neurons was significantly diminished as compared with AAV-transduced AGRP neurons (Figure 2D), that of AGRP in the ARC was drastically increased (Figure 2E). An increase in *Agrp* mRNA in the ARC of TET3-knockdown mice was also evident (Figure 2F), and the level of increase was comparable to that seen in fasted animals (Figure 1A). Importantly, AGRP neuron–specific TET3 knockdown did not affect AGRP neuronal viability (Figure 2G). We further confirmed the negative regulation of *Agrp* expression by TET3 using neuronal cell lines. As seen in Supplemental Figure 2A, siRNA-mediated TET3 knockdown in a mouse embryonic hypothalamic cell line led to increased *Agrp* expression. Likewise, TET3 knockdown using an siRNA specifically targeting human *TET3* (*TET3* siRNA) in a human neuronal cell line upregulated *AGRP* expression (Supplemental Figure 2B). Collectively, our results demonstrated that TET3 negatively regulated *Agrp* expression in AGRP neurons and that this regulation appeared to be conserved between mice and humans.

### TET3 knockdown activates AGRP neurons.

TET3 knockdown led to enhanced activity in AGRP neurons in brain slices isolated (9:00 am–10:00 am) from fed mice. The frequency of spontaneous action potentials (APs) was significantly higher (*t* test value [*t*] = 2.323, degrees of freedom [df] = 19, *P <* 0.05, 2-tailed Student’s *t* test) in ad libitum–fed knockdown mice (2.75 ± 0.84 Hz, *n =* 11 cells from 4 mice, Figure 2, H and I, red bar, left panel) than in controls (0.62 ± 0.28 Hz, *n =* 10 cells from 3 mice, Figure 2, H and I, blue bar, left panel). The AP threshold was –31.97 ± 2.57 mV (*n =* 11 cells from 4 mice, Figure 2I, red bar, right panel) in knockdown animals and –27.17 ± 3.21 (*n =* 10 cells from 3 mice, Figure 2I, blue bar, right panel) in controls, which was not significantly decreased (*t* = 1.177, df = 19, *P =* 0.25) (Figure 2I).

### Leptin fails to suppress fasting-induced overeating in TET3-knockdown mice.

**Figure 3:**
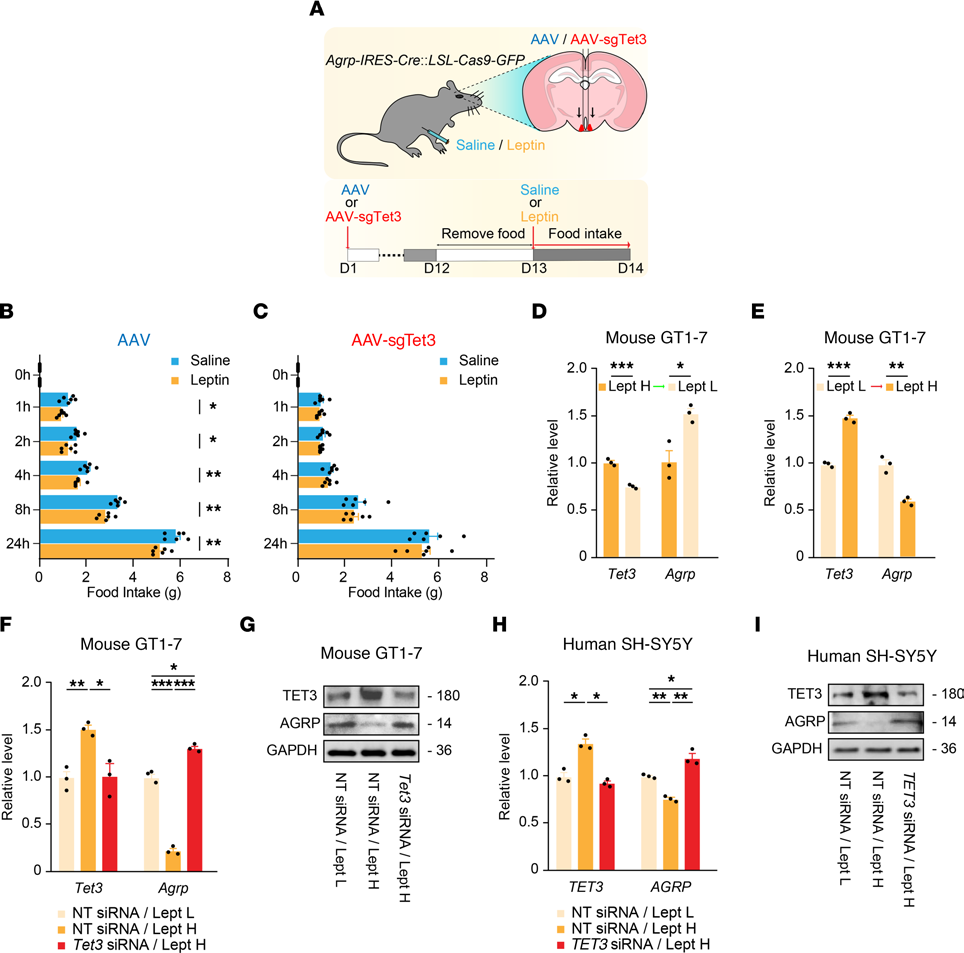
TET3 is required for leptin-induced repression of AGRP expression in cell lines. (**A**) Schematic diagram of a post-fast refeeding study of Cas9^+^ mice injected with AAV or AAV-sgTet3. (**B**) Food intake of mice injected with AAV at the indicated time points following administration of leptin or saline. *n* = 6 mice per group. *P < 0.05 and **P < 0.01, by 2-tailed Student’s *t* test. (**C**) Food intake of mice injected with AAV-sgTet3 following administration of leptin or saline. *n* = 6 mice per group. Significance was determined by 2-tailed Student’s *t* test. (**D**) Mouse GT1-7 cells maintained in a high leptin concentration (Lept H, 1 × 10^–8^ M) were switched to a low leptin concentration (Lept L, 1 × 10^–10^ M), followed by RNA extraction and qPCR of *Tet3* and *Agrp* mRNA 24 hours after the switch. *n =* 3 per group. *P < 0.05 and ***P < 0.001, by 2-tailed Student’s *t* test. (**E**) qPCR of *Tet3* and *Agrp* mRNA from GT1-7 cells maintained in Lept L and then switched to Lept H for 24 hours. *n* = 3 per group. **P < 0.01 and ***P < 0.001, by 2-tailed Student’s *t* test. (**F**) GT1-7 cells transfected with NT siRNA were maintained in Lept L (NT siRNA/Lept L) or Lept H (NT siRNA/Lept H) or were transfected with *Tet3* siRNA and maintained in Lept H (*Tet3* siRNA/Lept H). RNA was extracted 12 hours (for *Tet3*) or 36 hours (for *Agrp*) after the switch and analyzed by qPCR. *P < 0.05, **P < 0.01, and ***P < 0.001, by 1-way ANOVA with Tukey’s post test. (**G**) Representative immunoblots for TET3 and AGRP from GT1-7 cells treated as in **F**. GAPDH was used as a loading control. Proteins were isolated at the 36-hour time point. (**H**) Human SH-SY5Y neuroblastoma cells transfected with NT siRNA were maintained in Lept L (NT siRNA/Lept L) or Lept H (NT siRNA/Lept H) or were transfected with *TET3* siRNA and maintained in Lept H (*TET3* siRNA/Lept H). RNA was extracted 24 hours after the switch and analyzed by qPCR. *n* = 3 per group. *P < 0.05 and **P < 0.01, by 1-way ANOVA with Tukey’s post test. (**I**) Representative immunoblots for TET3 and AGRP from SH-SY5Y cells treated as in **H**. Proteins were isolated at the 36-hour time point. All data represent the mean ± SEM.

AGRP neuron activity is inhibited by the adipose hormone leptin (54). Given that CRISPR-mediated deletion of *Lepr* in adult AGRP neurons causes hyperphagia, obesity, and diabetes (15), we hypothesized that TET3 might affect leptin signaling in AGRP neurons. Thus, mice injected with AAV or AAV-sgTet3 were subjected to acute fasting followed by leptin or saline treatment and measurement of food intake (Figure 3A). While leptin suppressed hunger-induced appetite in the control mice (Figure 3B), it failed to do so in the TET3-knockdown animals (Figure 3C), suggesting that TET3 is necessary for leptin to inhibit hunger-induced overeating.

### TET3 mediates leptin-induced inhibition of AGRP expression in cell lines.

Circulating leptin levels fall during fasting or food deprivation (55–57). As food deprivation (i.e., low leptin signaling) downregulates TET3 in AGRP neurons (Figure 1C), we speculated that leptin might regulate TET3 expression. To test this possibility, we used neuronal cell lines. Thus, mouse GT1-7 hypothalamic cells maintained in a high leptin level were switched to a low leptin level (mimicking food deprivation), followed by gene expression analysis. Although the expression of *Agrp* expectedly increased, that of *Tet3* decreased in response to reduced leptin levels (Figure 3D). Conversely, when cells maintained in low leptin levels were switched to high leptin levels (mimicking refeeding), we obtained the opposite results (Figure 3E). These results suggested that leptin had a positive effect on *Tet3* expression. Next, when GT1-7 cells maintained in low leptin levels were switched to high leptin levels in the presence of TET3 knockdown (Figure 3F, left column), we found that *Agrp* expression no longer decreased in response to increased leptin at both mRNA (Figure 3F, right column) and protein (Figure 3G) levels. We also observed loss of leptin-induced inhibition of *AGRP* expression with TET3 knockdown in human neuronal cells (Figure 3, H and I). Collectively, these results showed that leptin upregulated TET3, which was required for leptin-induced inhibition of *Agrp/AGRP* expression both in mouse and human neuronal cells. Importantly, these results are in line with our in vivo findings that in fed mice, *Agrp* expression remained elevated in TET3-knockdown AGRP neurons (Figure 2, E and F).

### Mechanism of leptin-induced, TET3-dependent inhibition of Agrp expression.

**Figure 4:**
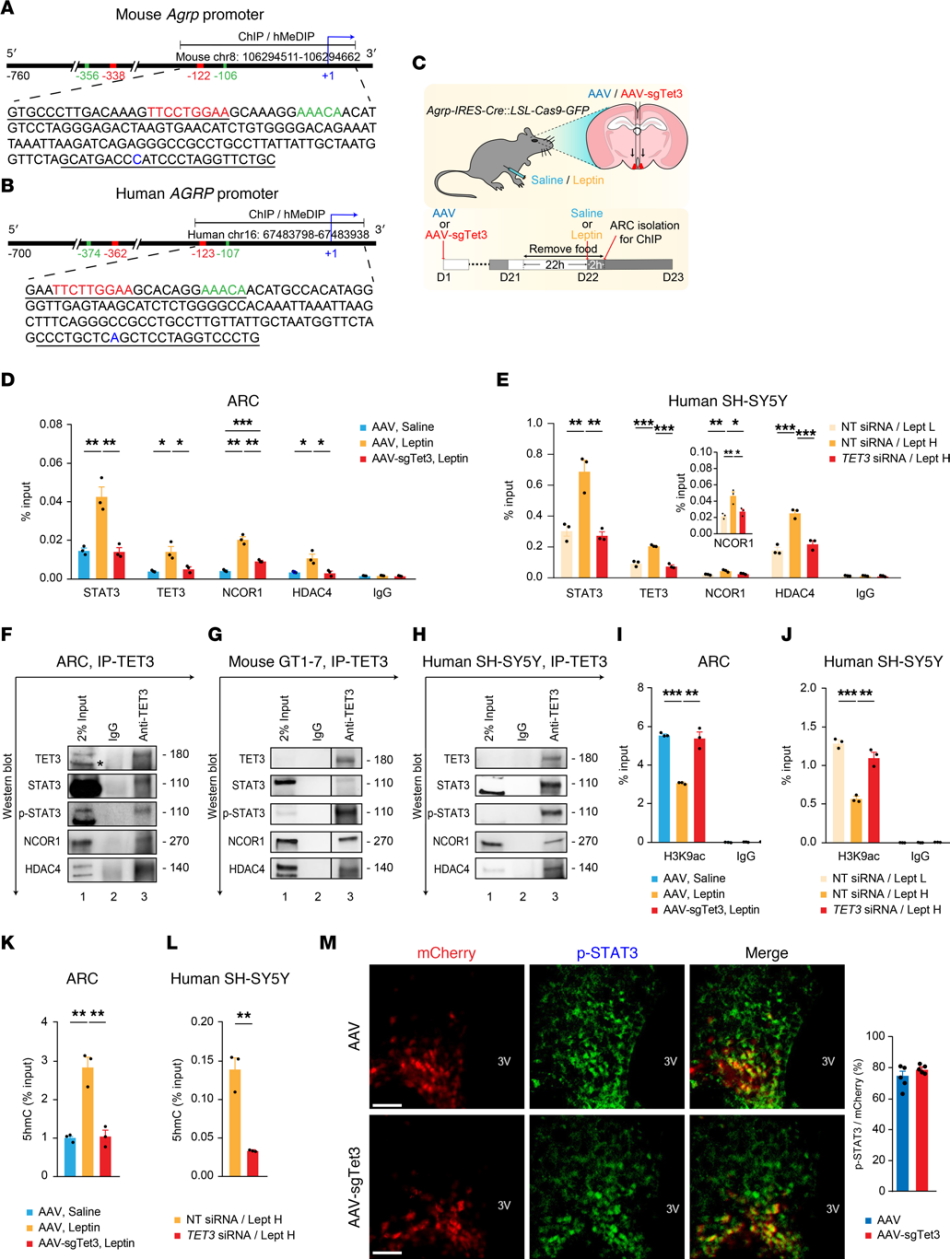
TET3 promotes the association of a chromatin-modifying complex with the *Agrp*/*AGRP* promoters. (**A** and **B**) STAT3- and FOXO1-binding sites labeled in red and green, respectively. Numbers depict starting and ending positions of nucleotides in chromosomes. PCR primers for the zoomed-in ChIP/hMeDIP regions are underlined. (**C**) Mice were injected with AAV-sgTet3 or AAV on day 1 (D1). On D21, the mice were fasted 22 hours and treated with leptin or saline 2 hours, followed by ARC isolation. (**D**) Mice were treated as in **C**. ARCs from 4 mice/group were pooled for ChIP-qPCR. *n* = 3/group, technical replicates. *P < 0.05, **P < 0.01, ***P < 0.001, 1-way ANOVA with Tukey post test. (**E**) SH-SY5Y cells transfected with NT siRNA were maintained in Lept L (NT siRNA/Lept L) or Lept H (NT siRNA/Lept H) or were transfected with *TET3* siRNA and maintained in Lept H (*TET3* siRNA/Lept H). Cells were collected 36 hours after transfection for ChIP-qPCR. Data presented as percentage of input. *n* = 3/group, technical replicates. *P < 0.05, **P < 0.01, ***P < 0.001, 1-way ANOVA with Tukey post test. (**F**) Mice were fasted 22 hours, followed by leptin injection and isolation of ARCs 2 hours after leptin injection. ARCs from 3 mice were pooled for co-IP. Representative immunoblots shown, protein sizes in kDa on right. The band labeled with an asterisk is likely an isoform of TET3. (**G**) Representative immunoblots of co-IP from GT1-7 cells maintained in Lept H. Samples in lanes 2 and 3 were run on the same gel but were noncontiguous. (**H**) Representative immunoblots of co-IP from SH-SY5Y cells maintained in Lept H. (**I**) Mice were treated as in **C**. ARCs from 2 mice/group were pooled for ChIP-qPCR. *n* = 3, technical replicates. **P < 0.01, ***P < 0.001, 1-way ANOVA with Tukey post test. (**J**) SH-SY5Y cells were treated as in **E**, followed by ChIP-qPCR. *n* = 3/group, technical replicates. **P < 0.01, ***P < 0.001, 1-way ANOVA with Tukey post test. (**K**) Mice were treated as in **C**. ARCs from 2 mice in each group were pooled for hMeDIP-qPCR. *n* = 3/group, technical replicates. **P < 0.01, 1-way ANOVA with Tukey post test. (**L**) hMeDIP-qPCR of SH-SY5Y cells transfected with NT siRNA or TET3 siRNA in Lept H 36 hours. *n* = 3/group, technical replicates. **P < 0.01, 2-tailed Student’s *t* tests. (**M**) Representative micrographs and statistical analysis of p-STAT3^+^ (green) AGRP neurons (red) from fed mice. *n* = 5/group. Two-tailed Student’s *t* test. Scale bars: 50 μm. Data represent mean ± SEM.

Binding of leptin to its receptor in AGRP neurons activates JAK2 which phosphorylates STAT3 at Tyr705 (p-STAT3); p-STAT3 then migrates as a dimer to the nucleus, where it inhibits transcription of both *Agrp* and *Npy* (14). However, the molecular mechanism underpinning this transcriptional regulation is complex and has remained incompletely understood. A large region of DNA (42.5 kb) upstream of the transcriptional start site of the mouse *Agrp* gene was identified to be both necessary and sufficient for the spatial expression and fasting response of AGRP in transgenic mice (58). This regulatory region includes an evolutionarily conserved proximal promoter of 760 bp (Figure 4A) that overlaps with a minimal promoter of 700 bp of human *AGRP* (Figure 4B) (58, 59). Using in vitro gel shift and luciferase reporter assays, 2 STAT3-binding sites and 2 FOXO1-binding sites adjacent to each other were identified in the mouse *Agrp* promoter (60) (Figure 4A). These studies also identified 1 STAT3-binding site adjacent to 1 FOXO1-binding site in the promoter of mouse *Pomc*, which is exclusively expressed in POMC neurons (60). Treating primary cells isolated from mouse hypothalami (which contain mixed populations of AGRP and POMC neurons and other cell types) with leptin induced binding of STAT3 and inhibited binding of FOXO1 to these sequences (60). Using in vivo non–cell type–specific approaches (i.e., ARC injection of adenoviral expression vectors), STAT3 and FOXO1 were found to elicit opposing actions on the expression of *Agrp* and *Pomc* in mice, with STAT3 inhibiting and FOXO1 activating *Agrp* and FOXO1 inhibiting and STAT3 activating *Pomc* (60). Given the non–cell type–specific nature of these studies (60), a clear mechanistic understanding of leptin-induced, STAT3-mediated repression of *Agrp* expression in AGRP neurons is still lacking.

Gene expression is strongly influenced by the accessibility of nucleosomal DNA and the state of chromatin compaction. Histone acetylation plays key roles in modulating chromatin structure and function. While acetylation is generally associated with an open chromatin state and active transcription, deacetylation is associated with transcriptional repression. Histone acetyltransferases (HATs) and histone deacetylases (HDACs) act antagonistically to control histone acetylation (61). Transcriptional coregulators (including both coactivators and corepressors) act to bridge transcription factors and chromatin-modifying enzymes such as HATs and HDACs, determining the final transcriptional output. The same transcription factors can elicit opposing effects depending on which coregulators they interact with. NCOR1 is among the best-characterized corepressors shown to inhibit transcription by recruiting various HDACs in a context-specific manner (62).

We hypothesized that, in response to increased leptin levels, TET3, via interaction with STAT3, targets a transcriptional corepressor complex containing NCOR1 and HDAC4 to *Agrp* to inhibit transcription. First, TET3 is required for leptin-induced repression of *Agrp* expression (Figure 3, F–I). Second, as a nonspecific DNA-binding protein, TET3 is targeted to specific genomic loci via interaction with transcription factors (27, 63). Third, STAT3 is a transcription factor shown to physically interact with TET3 in human glioma cells (64). Fourth, previous in vitro studies showed that STAT3 and FOXO1 compete for binding to the mouse *Agrp* promoter and that decreasing expression of FOXO1 induces binding of NCOR1 to the *Agrp* promoter (60). Fifth, HDAC4 was found to be exclusively localized to the nuclei of mouse AGRP neurons by immunofluorescence (65). Finally, mutations in the *HDAC4*/*Hdac4* genes have been associated with eating disorders in both humans and mice (66, 67). To test our hypothesis, we first asked whether we could detect binding of STAT3, TET3, NCOR1, and HDAC4 to the mouse *Agrp* promoter at the basal level (in a fasted state when leptin levels are low) and, if so, whether the binding would be affected by leptin treatment. Thus, mice injected with AAV or AAV-sgTet3 bilaterally into the ARC were fasted and treated with leptin or saline, followed by isolation of ARCs and ChIP-qPCR analysis (Figure 4C). While leptin increased binding of STAT3, TET3, NCOR1, and HDAC4 to the *Agrp* promoter as compared with basal, it failed to do so in AAV-sgTet3–injected mice (Figure 4D). Notably, in these mice the association of STAT3, NCOR1, and HDAC4 with the promoter remained at the basal level after leptin treatment (Figure 4D), suggesting that TET3 was required for leptin-induced association of these proteins with the promoter. Importantly, this leptin-induced, TET3-dependent association of STAT3, NCOR1, and HDAC4 with the *Agrp* promoter in mice was recapitulated in human neuronal cells (Figure 4E), suggesting a conserved mechanism.

To provide further evidence supporting this hypothesis, we performed co-IP studies to examine protein-protein interactions in the presence of leptin. Thus, mice were fasted and then treated with leptin for 2 hours, followed by ARC isolation and co-IP studies. When TET3 was pulled down (Figure 4F, top blot, lane 3), STAT3/p-STAT3 (second and third blots from top, lane 3), NCOR1 (fourth blot from top, lane 3), and HDAC4 (bottom blot, lane 3) were detected in the immunoprecipitated complexes. We obtained similar results using mouse and human neuronal cells (Figure 4, G and H). Both STAT3 and HDAC4 are known to undergo a variety of posttranslational modifications including phosphorylation, acetylation, and methylation in a context-dependent manner (68, 69), which can affect protein mobility on Western blot gels. The enrichment of p-STAT3 (phosphorylated at Tyr705) in TET3-containing complexes both in vivo (Figure 4F) and in vitro (Figure 4, G and H) was consistent with a functional interaction between TET3 and p-STAT3. Taken together with our ChIP data (Figure 4, D and E), these results suggested that TET3, p-STAT3, NCOR1, and HDAC4 probably formed a multiprotein complex on the *Agrp*/*AGRP* promoters.

To determine whether the leptin-induced, TET3-dependent association of the corepressor complex affects histone acetylation, we performed ChIP-qPCR analysis using an antibody specific for H3K9ac, a histone mark for active transcription (61). Thus, mice were treated as in Figure 4C, and ChIP experiments were performed using anti-H3K9ac. We observed a leptin-induced reduction in histone acetylation at the *Agrp* promoter, which was abolished in TET3-knockdown mice (Figure 4I). This leptin-induced, TET3-dependent reduction in histone acetylation at the *AGRP* promoter was also observed in human neuronal cells (Figure 4J). Collectively, our results suggested that leptin induced the formation of a transcriptional corepressor complex on the *Agrp*/*AGRP* promoters in a TET3-dependent manner, leading to histone deacetylation and inhibition of transcription.

### TET3 induces 5hmC modification of the Agrp/AGRP promoters.

**Figure 5:**
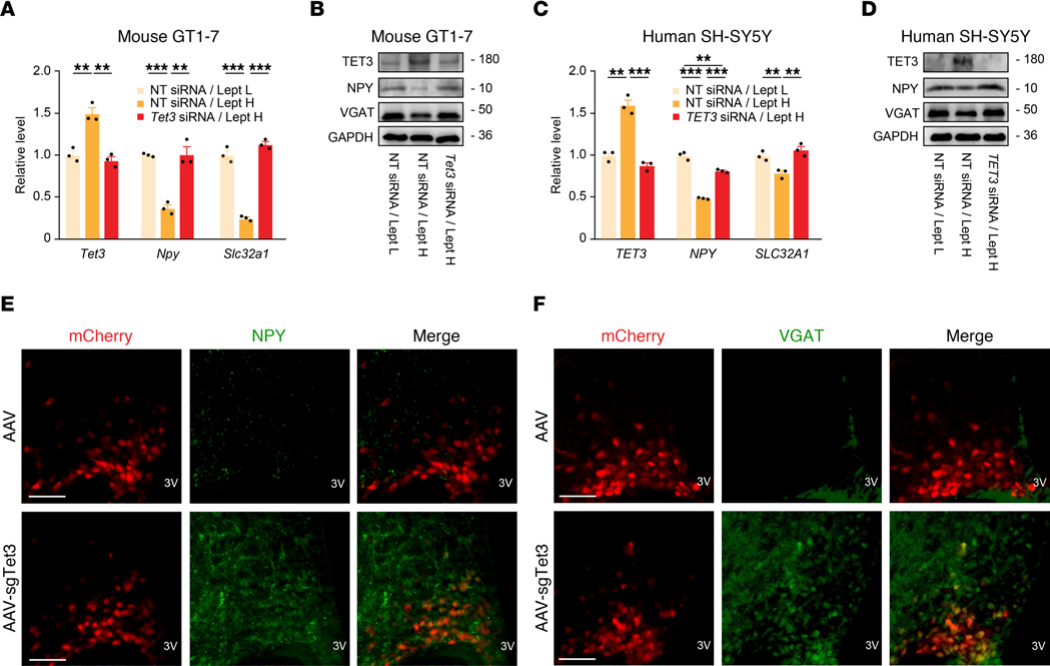
TET3 negatively regulates the expression of NPY and VGAT. (**A**) GT1-7 cells transfected with NT siRNA were maintained in Lept L (NT siRNA/Lept L) or Lept H (NT siRNA/Lept H) or were transfected with *Tet3* siRNA and maintained in Lept H (*Tet3* siRNA/Lept H). RNAs were extracted at 12 hours (for *Tet3*) or 36 hours (for *Npy* and *Slc32a1*) following the switch and analyzed by qPCR. *n* = 3 per group in technical replicates. **P < 0.01 and ***P < 0.001, by 1-way ANOVA with Tukey’s post test. (**B**) Representative immunoblots for TET3, NPY, and VGAT from GT1-7 cells treated as in **A**. Proteins were isolated at the 36**-**hour time point. (**C**) SH-SY5Y cells transfected with NT siRNA were maintained in Lept L (NT siRNA/Lept L) or Lept H (NT siRNA/Lept H) or were transfected with *TET3* siRNA and maintained in Lept H (*TET3* siRNA/Lept H). RNA was extracted at 12 hours (for *TET3*) or 36 hours (for *NPY* and *SLC32A1*) following the switch and analyzed by qPCR. *n* = 3 per group in technical replicates. **P < 0.01 and ***P < 0.001, by 1-way ANOVA with Tukey’s post test. (**D**) Representative immunoblots for TET3, NPY, and VGAT from SH-SY5Y cells treated as in **C**. Proteins were isolated at the 36-hour time point. (**E** and **F**) Representative micrographs of NPY (green) and VGAT (green) in the ARCs of Cas9^+^ mice injected with AAV or AAV-sgTet3. AGRP neurons from the injected viruses are labeled red. Scale bars: 50 μm. All data represent the mean ± SEM.

TETs initiate DNA demethylation by oxidizing 5mC to 5hmC; 5hmC also serves as a stable epigenetic mark. In postmitotic neurons, 5hmC accumulates to approximately 10 times the levels present in peripheral cell types (70–72). 5mC and 5hmC have been detected at both CpG and non-CpG (CpH, where H = A/C/T) dinucleotides (30, 73, 74) and can function to enhance or inhibit the binding of regulatory protein factors in a context-dependent manner (29, 30). In postmitotic neurons, non-CpG 5hmC occurs predominantly in CpA dinucleotides (30). We noticed an enrichment of CpA dinucleotides in both the mouse and human *Agrp*/*AGRP* promoters (Figure 4, A and B). We hypothesized that binding of TET3 to the *Agrp*/*AGRP* promoters induces 5hmC modification, enabling a stable association of STAT3 and the corepressor complex with the promoters. Thus, mice were treated as in Figure 4C, and genomic DNA was isolated from the ARCs and subjected to hydroxymethylated DNA IP–qPCR (hMeDIP-qPCR) analysis as previously described (48). As seen in Figure 4K, an increase in 5hmC in the *Agrp* promoter in leptin- versus saline-treated mice was evident, whereas in TET3-knockdown animals, no increase in 5hmC was detected after leptin treatment. Thus, a positive correlation exists between TET3 binding (Figure 4D) and 5hmC modification (Figure 4K) of the *Agrp* promoter in vivo. Importantly, the TET3-dependent increase in 5hmC modification of the *AGRP* promoter was recapitulated in human cells (Figure 4L). Further, TET3 knockdown did not affect STAT3 phosphorylation in AGRP neurons in mice (Figure 4M), suggesting that the reduced association of STAT3 with the *Agrp* promoter seen in TET3-knockdown AGRP neurons (Figure 4D) was not a result of decreased STAT3 phosphorylation. Given the positive connection between 5hmC (Figure 4, K and L) and binding of STAT3 and the corepressor complex to the *Agrp*/*AGRP* promoters (Figure 4, D and E), we propose that TET3-induced 5hmC modification in the *Agrp*/*AGRP* promoters enables a stable association of STAT3 and the corepressor complex with the promoters.

### TET3 negatively affects the expression of Npy and Slc32a1.

Activated AGRP neurons release AGRP, NPY, and GABA, which act in concert to stimulate food intake and reduce energy expenditure. We sought to test whether the expression of *Npy* and *Slc32a1* (encoding VGAT, which is required for loading GABA into synaptic vesicles) might also be regulated by TET3. Unlike AGRP, which is expressed exclusively in AGRP neurons, both NPY and VGAT are also expressed in other neurons. Similar to what we observed for AGRP (Figure 3, F–I), leptin negatively affected the expression of NPY and VGAT in a TET3-dependent manner, at both the mRNA and protein levels (Figure 5, A and B). This regulation was also observed in human cells (Figure 5, C and D). Further, we detected a significant increase in the protein signals of NPY and VGAT in the ARCs of TET3-knockdown mice compared with control mice (Figure 5, E and F), consistent with TET3-dependent inhibition of NPY and VGAT expression in AGRP neurons. The diffuse signals of NPY and VGAT reflect NPY being a secreted peptide and VGAT being in synaptic vesicles of neuronal projections. Further, we observed increased expression of *Agrp* mRNA and decreased expression of *Pomc* mRNA in the ARCs of ad libitum–fed mice injected with AAV-sgTet3 versus ARCs of mice injected with AAV (Supplemental Figure 3), consistent with the notion that activated AGRP neurons inhibit POMC neurons through the release of GABA (3).

### AGRP neuron–specific TET3 knockdown causes hyperphagia, obesity, and diabetes.

**Figure 6:**
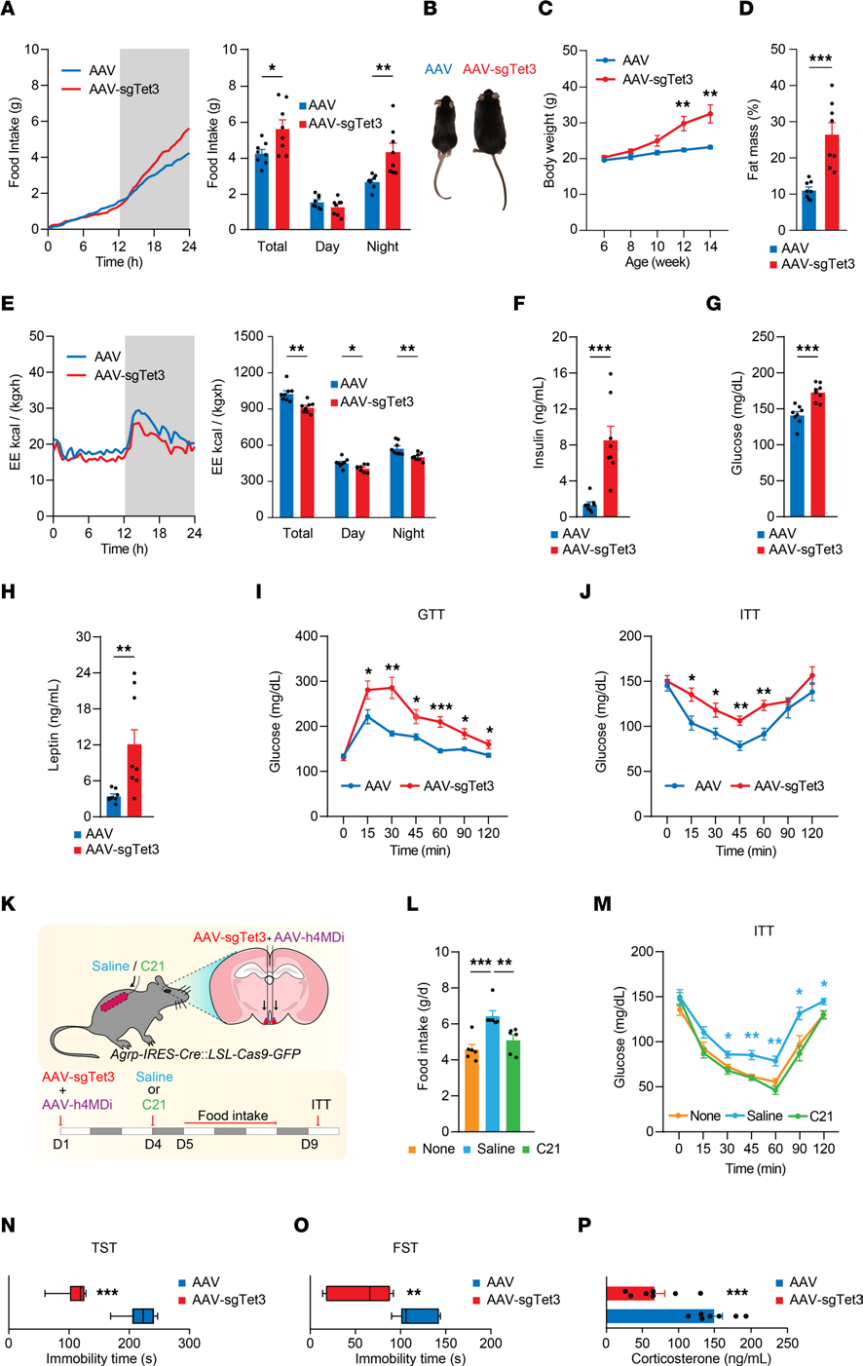
TET3 knockdown in AGRP neurons in female mice induces hyperphagia, obesity, and diabetes and reduces stress-like behaviors. (**A**) Cas9^+^ mice injected with AAV-sgTet3 or AAV bilaterally into the ARC at the age of 6 weeks became hyperphagic 2 weeks after injection. *n* = 8 animals per group. *P < 0.05 and **P < 0.01, by 2-tailed Student’s *t* test. (**B**) Representative images of mice 8 weeks after injection. (**C**) Post-injection body weight changes in mice. *n* = 8 animals per group. **P < 0.01, by 2-tailed Student’s *t* test. (**D**) Fat mass in mice 8 weeks after injection. *n* = 8 animals per group. ***P < 0.001, by 2-tailed Student’s *t* test. (**E**) Energy expenditure (EE) 2 weeks after injection. *n* = 8 animals per group. *P < 0.05 and **P < 0.01, by 2-tailed Student’s *t* test. (**F**) Blood insulin levels in ad libitum–fed mice 5 weeks after injection. *n* = 8 animals per group. ***P < 0.01, by 2-tailed Student’s *t* test. (**G**) Blood glucose levels in ad libitum–fed mice 6 weeks after injection. *n* = 8 animals per group. ***P < 0.01, by 2-tailed Student’s *t* test. (**H**) Blood leptin levels in ad libitum–fed mice 7 weeks after injection. *n =* 8 animals per group. **P < 0.01, by 2-tailed Student’s *t* test. (**I**) Glucose tolerance test (GTT) results for mice 8 weeks after injection. *n =* 8 animals per group. *P < 0.05, **P < 0.01, and ***P < 0.01, by 2-way ANOVA with Šidák’s post test. (**J**) Insulin tolerance test (ITT) results for mice 9 weeks after injection. *n* = 8 animals per group. *P < 0.05 and **P < 0.01, by 2-way ANOVA with Šidák’s post test. (**K**) Schematic diagram of experiments. Cas9^+^ mice were coinjected with AAV-sgTet3 and AAV-hM4Di bilaterally into the ARC on day 1 (D1), followed by implantation of an osmotic pump containing saline or C21 on day 4. Food intake measurements and ITTs were performed on day 5 and day 9, respectively. (**L**) Food intake data. “None” indicates age-matched Cas9^+^ mice without AAV injection or osmotic pump. *n =* 6 animals per group. **P < 0.01 and ***P < 0.001, by 1-way ANOVA with Tukey’s post test. (**M**) ITT data. *n* = 6 animals per group. *P < 0.05 and **P < 0.01, by 1-way ANOVA with Tukey’s post test. (**N**) Tail suspension test (TST) immobility scores for Cas9^+^ mice injected with AAV or AAV-sgTet3. *n* = 7 animals per group. ***P < 0.001, by 2-tailed Student’s *t* test. (**O**) Forced swim test (FST) immobility scores of Cas9^+^ mice injected with AAV or AAV-sgTet3. *n* = 7 animals per group. **P < 0.01, by 2-tailed Student’s *t* test. (**P**) Plasma corticosterone concentrations in Cas9^+^ mice injected with AAV or AAV-sgTet3. *n* = 7 animals per group. ***P < 0.001, by 2-tailed Student’s *t* test. All data represent the mean ± SEM.

Activated AGRP neurons can release AGRP, NPY, GABA, and augmentor α, all of which individually and in concert can potently affect feeding and systemic glucose metabolism (75–79). Notably, deletion of VGAT, which is required for vesicular loading of GABA, leads to complete loss of synaptic GABA release from AGRP neurons (6, 7). In line with these observations, CRISPR-mediated TET3 knockdown in AGRP neurons induced hyperphagia, obesity, and diabetes, as determined by increased food intake (Figure 6A), increased body weight and fat mass (Figure 6, B–D), decreased energy expenditure (Figure 6E), elevated blood insulin, glucose, and leptin levels (Figure 6, F–H), and decreased glucose tolerance and insulin sensitivity (Figure 6, I and J), in both female (Figure 6, A–J) and male (Supplemental Figure 4) mice. Notably, the increases in food intake (Figure 6A, and Supplemental Figure 4A) and energy expenditure (Figure 6E and Supplemental Figure 4E) in AAV-sgTet3–injected mice were observed 2–3 weeks after injection, before significant increases in body weight/fat mass could be detected (Figure 6C and Supplemental Figure 4C), suggesting direct and body weight/fat mass–independent effects of TET3 knockdown in AGRP neurons.

To determine the relevance of activated AGRP neurons following TET3 knockdown, we used the chemogenetic tool of designer receptors exclusively activated by designer drugs (DREADDs). We coinjected AAV-sgTet3 with an AAV containing a Cre-dependent hM4Di-mCherry transgene (AAV-hM4Di) (53) bilaterally into the ARC of Cas9^+^ mice, followed by implantation of an osmotic pump to infuse the DREADD agonist 21 (C21) (80) or saline (Figure 6K). We confirmed AGRP neuron–specific expression of hM4Di by immunofluorescence (Supplemental Figure 5). Stimulation of hM4Di with C21, which thereby inhibited AGRP neurons, suppressed hyperphagia and reversed systemic insulin resistance induced by TET3 knockdown in ad libitum–fed mice as early as 1 week after injection (Figure 6, L and M). These results showed that activation of AGRP neurons as a result of TET3 knockdown contributed to both hyperphagia and systemic insulin resistance.

### AGRP neuron–specific TET3 knockdown reduces stress-like behaviors.

We and others have previously shown that activation of AGRP neurons affects many complex behaviors beyond feeding (18, 19, 21, 81). The melanocortin system, to which AGRP belongs, has also been tied to stress and depression (81, 82). Because TET3 knockdown in AGRP neurons activated these cells, we evaluated stress-like behaviors in these animals using a tail suspension test and a forced swim test. The TET3-knockdown animals had shorter immobility times than did the control mice in both the tail suspension test (Figure 6N) and the forced swim test (Figure 6O), indicating decreased stress-like states. Further, compared with control animals, the TET3-knockdown mice had reduced plasma cortisol levels (Figure 6P). Collectively, these data suggested that TET3 knockdown in AGRP neurons produced anti-stress effects.

## Discussion

**Figure 7:**
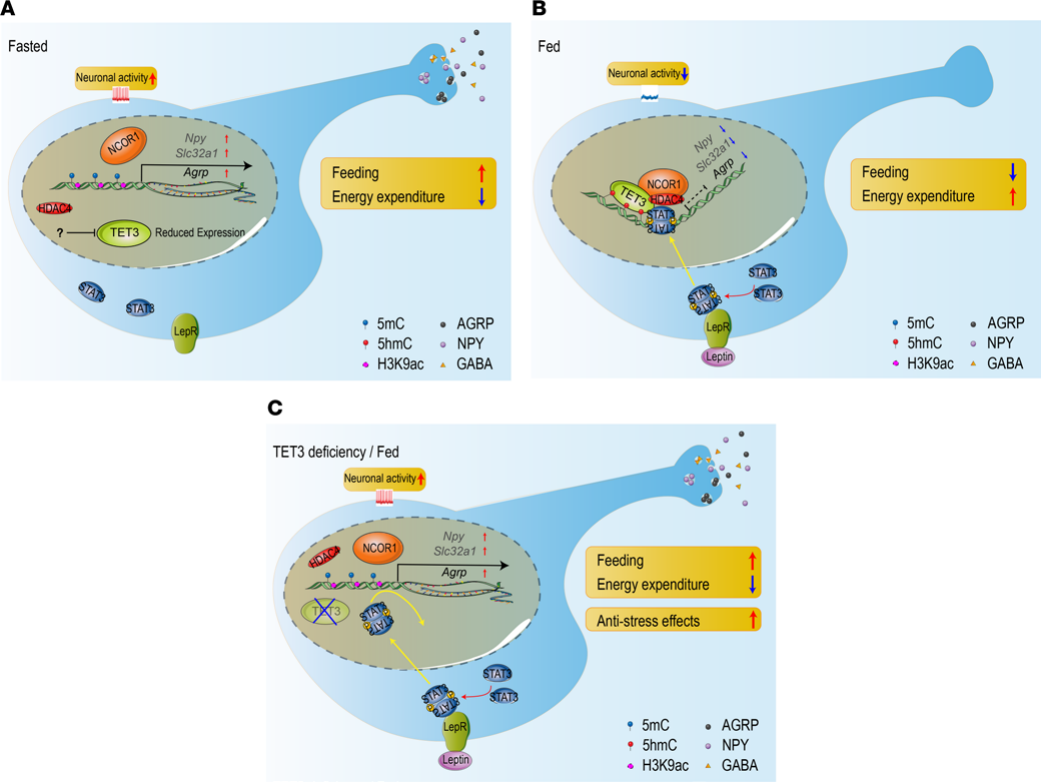
A proposed model. (**A**) In a fasted state, leptin signaling and TET3 levels are low, and there is no association of the chromatin-modifying complex with the *Agrp* promoter. Histones are acetylated and the chromatin is in an open state, *Agrp* transcription is on, and the neurons are active. The lack of inhibition of expression of *Agrp*, *Npy*, and *Slc32a1* by TET3 enables sustained production and synaptic release of AGRP, NPY, and GABA. The physiological outcomes are increased food intake and decreased energy expenditure. (**B**) In a fed state, a rise in leptin levels promotes binding of p-STAT3 to the *Agrp* promoter, which in turn recruits TET3 and the chromatin-modifying complex. Binding of TET3 induces 5hmC modification, which is required for a stable association of STAT3 and the chromatin-modifying complex with the promoter. The chromatin-modifying complex promotes histone deacetylation, thereby inducing a closed chromatin state and inhibition of *Agrp* transcription. Neuronal activity is also suppressed, in part, by a yet-unknown TET3-mediated mechanism. The expression of all 3 genes is reduced. The physiological outcomes are decreased food intake and increased energy expenditure. (**C**) In a fed state without TET3 expression, there is no 5hmC modification, activated STAT3 is unable to stably associate with the *Agrp* promoter to allow recruitment of the chromatin-modifying complex, histones remain acetylated, the chromatin is open, and *Agrp* transcription is not inhibited. In addition, the neuron remains active due to the lack of inhibition from a yet-unidentified TET3-dependent mechanism. The lack of inhibition of expression of all 3 genes enables sustained production and synaptic release of AGRP, NPY, and GABA. The physiological outcomes are increased food intake and decreased energy expenditure. The sustained neuronal activity and synaptic release of AGRP, NPY, and GABA also promote anti-stress effects.

In the present study, we found that food deprivation downregulated TET3 in AGRP neurons and that CRISPR-mediated, cell-specific ablation of TET3 in AGRP neurons activated these neurons and upregulated the expression of *Agrp*, *Npy*, and *Slc32a1* in adult mice. We also found that AGRP neuron–specific TET3 knockdown caused hyperphagia, obesity, and diabetes in both female and male mice, highlighting a central role of TET3 in regulating feeding, body weight, and glucose metabolism by AGRP neurons. Using both mouse models and human and mouse neuronal cell lines, we demonstrated that TET3 knockdown in AGRP neurons dysregulated neuronal activity and impaired leptin signaling. In particular, we revealed that TET3 was required for leptin-induced inhibition of *Agrp*/*AGRP* expression both by promoting 5hmC modification and by recruiting a chromatin-modifying complex to the promoters of *Agrp*/*AGRP*, and that this mechanism of dual action of TET3 appeared to be conserved between mice and humans. Furthermore, we showed that TET3 knockdown in AGRP neurons induced anti-stress effects. On the basis of our findings, we propose the model illustrated in Figure 7.

While our work has uncovered an important aspect of TET3 regulation as a critical epigenetic component of the neural circuits in the control of satiety, energy metabolism, and nonfeeding behaviors, the current studies have a number of limitations. First, although the requirement of STAT3 in leptin signaling has been well established, our data showing that STAT3, TET3, NCOR1, and HDAC4 formed a multiprotein complex on the *Agrp/AGRP* promoters suggest that NCOR1 and HDAC4 may be important for leptin signaling, although whether they are required for leptin effects remains to be determined. Second, FOXO1 has been suggested to compete with STAT3 for binding to the *Agrp* promoter, stimulating transcription (60). What role FOXO1 might play in the TET3-dependent regulation of *Agrp* expression in AGRP neurons warrants future investigation. Third, given the robust metabolic phenotype of TET3 knockdown in AGRP neurons, it is quite likely that *Agrp*, *Npy*, and *Slc32a1* are not the only genes regulated by TET3. Indeed, TET3 deficiency caused chronic activation of AGRP neurons independent of food or energy status (this study), suggesting other yet-unidentified genes affected by TET3. In addition, it has been previously reported that leptin failed to elicit acute suppression of hunger-induced overeating in mice with AGRP neuron–specific disruption of GABA_A_ receptors. This phenotype was recapitulated by dorsomedial hypothalamic nucleus (DMH) neuron–specific deletion of *Lepr*, due, at least in part, to the loss of GABAergic afferents on AGRP neurons that are necessary for AGRP neurons to inhibit appetite. Under normal conditions, leptin acts on its receptor in DMH neurons to promote the release of GABA, which in turn acts on AGRP neurons, enabling them to suppress food intake (15). We found that AGRP neuron–specific TET3 knockdown abolished leptin’s ability to suppress fasting-induced overeating, raising an intriguing possibility that TET3 may directly or indirectly regulate the expression of genes encoding GABA_A_ receptors. Further, in cultured hippocampal neurons, TET3 acts as a synaptic sensor in the regulation of neuronal activity: increased synaptic activity upregulates TET3 expression, whereas TET3 inhibition elevates excitatory glutamatergic synaptic transmission (83). Likewise, CRISPR-mediated TET3 deletion in young mice increases excitatory and decreases inhibitory synaptic transmission in cerebral cortex neurons (84). Future studies aimed at identifying other TET3 targets as well as a more in-depth dissection of protein-protein interactions of TET3, STAT3, NCOR1, HDAC4, and FOXO1 are needed to obtain a more comprehensive mechanistic understanding of TET3 in the central regulation of feeding and energy metabolism, including its involvement with the known role of synaptic plasticity in these circuits (85, 86). Finally, although cell lines allow for a better dissection of certain molecular and cellular events that cannot be teased apart in whole organismal models, the derived results may not perfectly recapitulate in vivo conditions. Thus, we must interpret our cell line data with caution and in the context of in vivo studies.

The essential role of AGRP neurons in the regulation of food intake, body weight, and energy metabolism has been unambiguously established (4, 5). We believe our discovery of TET3 as an essential mediator of leptin-induced suppression of *Agrp* expression in AGRP neurons is conceptually novel. First, none of the TET family proteins has been previously documented to play a role in the central control of feeding, obesity, and glucose metabolism. Second, the current understanding has focused on the notion that leptin signaling activates STAT3, which binds to the *Agrp* promoter and inhibits transcription. However, how inhibition of transcription is accomplished has not been well defined. We showed that leptin-induced STAT3 binding enabled TET3-dependent recruitment of the transcriptional corepressor sNCOR1 and HDAC4 to the *Agrp*/*AGRP* promoters, which in turn promoted histone deacetylation, leading to inhibition of transcription. Third, as 5hmC modification of DNA is known to affect protein binding (29, 30), our results suggested that TET3-induced 5hmC modification of the *Agrp*/*AGRP* promoters enabled a stable association of STAT3 and a chromatin-modifying complex with the promoters and that TET3 knockdown did not alter STAT3 phosphorylation. As dysregulation of leptin signaling is tightly associated with human obesity and diabetes (10), our discovery of a dual action of TET3 (5hmC modification and recruitment of chromatin-modifiers) in the regulation of *Agrp*/*AGRP* expression in both human and mouse cells offers new opportunities for the future development of therapeutic interventions for metabolic disorders and related psychiatric conditions.

## Methods

See the Supplemental Methods for a detailed description of additional materials and methods.

### Immunofluorescence.

Postfixed sections were cut into 40 μm thick sections. After 10 minutes of washing 5 times in wash buffer (0.1 M PB, 0.4% Triton X-100, 1% BSA, 0.1 l-lysine, pH 7.3–7.5), the sections were incubated in blocking solution (1:50 normal donkey serum in washing buffer) for 20 minutes at room temperature. Sections were incubated with anti-TET3 (dilution 1:2,000; ABE290, MilliporeSigma) (48) (Supplemental Figure 1), anti-AGRP (dilution 1:2,000; PA5-78739, Invitrogen, Thermo Fisher Scientific; validated by the vendor), anti–p-STAT3 (Tyr705) (dilution 1:2,000; Cell Signaling Technology, 9145S) (87), anti-NPY (dilution 1:800, Cell Signaling Technology, 11976S) (88), or anti-VGAT (dilution 1:200, Abcam, Ab235952; validated by the vendor) overnight at 4°C. Negative control experiments were performed by omitting the respective primary antibodies. The next day, sections were washed 5 times (15 minutes each) in PBS and incubated in 0.4% Triton X-100 PBS with the following respective secondary antibodies for 1 hour at room temperature: donkey anti–rabbit IgG Fluor 350 (dilution 1:500, A10039, Invitrogen, Thermo Fisher Scientific) and donkey anti–rabbit IgG Fluor 594 (dilution 1:500, A-21207, Invitrogen, Thermo Fisher Scientific). The sections were coverslipped and visualized using a Keyence BZ-X700 fluorescence microscope. The fluorescence signals from GFP and mCherry in AGRP neurons were detected without immunostaining.

### Neuronal cell culture and treatments.

The mouse GT1-7 hypothalamic neuronal cell line (MilliporeSigma, SCC116), the human SH-SY5Y neuronal blastoma cell line (American Type Culture Collection [ATCC], CRL-2266), and the embryonic mouse hypothalamus cell line N11 (mHypoE-N11) (Cedarlane, CLU107) were purchased and cultured according to the manufacturers’ instructions. For siRNA transfection in a 24-well plate scale, cells were seeded at a density of 2 × 10^5^ cells/well the day before transfection. To prepare siRNA transfection solution for each well of cells, 5 pmol NT siRNA (nontargeting control siRNA, AM4636, Ambion), *Tet3* siRNA (siRNA specifically targeting mouse *Tet3*, 4390815/s101483, Ambion), or *TET3* siRNA (siRNA specifically targeting human *TET3*, 4392420/s47238, Ambion) (89, 90) was mixed with 25 μL OPTI-MEM (Gibco, Thermo Fisher Scientific, 31985-070) by gentle pipetting. In parallel, 1.5 μL Lipofectamine RNAiMAX (Invitrogen, Thermo Fisher Scientific, 13778-150) was mixed with 25 μL OPTI-MEM by gentle pipetting. After 5 minutes of incubation at room temperature, the resulting 50 μL transfection solution was added to 1 well of cells containing 1 mL culture media. For the GT1-7 transfection shown in Supplemental Figure 1, B and C, and the mHypoE-N11 transfection shown in Supplemental Figure 2A, the media were changed the next day, followed by RNA extraction or immunofluorescence 48 hours after transfection. In the experiments shown in Supplemental Figure 1, B and C, and Supplemental Figure 2A, no leptin was present in the culture media. For leptin treatments, cells were incubated with leptin (mouse leptin L3772-1MG, MilliporeSigma, for GT1-7; human leptin L4146-1MG, MilliporeSigma, for SH-SY5Y) at concentrations of 1 × 10^–8^ M (Lept H) or 1 × 10^–10^ M (Lept L) in culture media. The durations of Lept H and Lept L treatments are indicated in the figure legends.

### ChIP-qPCR.

To prepare antibodies, 5 μL (packed volume) ChIP-grade Dynabeads Protein G (Invitrogen, Thermo Fisher Scientific, 10004D) was washed twice with 1 mL binding buffer (0.2% Tween 20 in PBS), followed by incubation on a rotator with 10 μg rabbit polyclonal anti-TET3 (Active Motif, 61395) (Supplemental Figure 6A), anti-STAT3 (Proteintech, 10253-2-AP) (91), anti-NCOR1 (Cell Signaling Technology, 5948S) (92), anti-HDAC4 (Active Motif, 40969) (Supplemental Figure 6B), anti-H3K9ac (Active Motif, 39137) (validated by the vendor), or preimmune rabbit IgG (as a negative control) in 350 μL binding buffer at 4°C overnight. Antibody-bound beads were washed twice with 1 mL binding buffer, resuspended in 50 μL dilution buffer (0.01% SDS, 1.1% Triton X-100, 1.2 mM EDTA, 16.7 mM Tris-HCl at PH 8.0, 167 mM NaCl), and kept on ice until use. To prepare chromatin, freshly isolated ARCs (2 ARCs from 1 mouse per ChIP, Figure 4, D and I) were washed twice with 1 mL cold-PBS, followed by cross-linking in 1% paraformaldehyde/PBS on a rotator at room temperature for 15 minutes. Glycine buffer (150 mM final concentration) was added and incubated in rotation at room temperature for 10 minutes to quench cross-linking. Cross-linked ARCs were washed twice with PBS and homogenized (5–10 strokes) using a disposable pellet pestle (Thermo Fisher Scientific, 12-141-368) in 300 μL cold cell lysis buffer (50 mM Tris-HCl at pH 8.0, 140 mM NaCl, 1 mM EDTA, 10% glycerol, 0.5% NP-40, 0.25% Triton X-100), followed by incubation at 4°C for 20 minutes to lyse the plasma membrane. For cell ChIP experiments (Figure 4, E and J), SH-SY5Y cells seeded at a density of 6 × 10^6^ cells/plate in a 100 mm plate the night before were transfected with nontargeting (NT) siRNA in Lept L (NT siRNA/Lept L) or Lept H (NT siRNA/Lept H), or with *TET3* siRNA in Lept H. After 48 hours, 360 μL 32% paraformaldehyde (final concentration = 1%) was added to the plate to cross-link cells at room temperature for 10 minutes, followed by addition of glycine buffer (150 mM final concentration) to quench cross-linking for 5 minutes. Cross-linked cells were washed twice with PBS and harvested in 1,000 μL cold cell lysis buffer, followed by incubation at 4°C for 20 minutes to lyse the plasma membrane. Nuclei from ARCs or SH-SY5Y cells were pelleted by centrifugation at 2,000*g* for 5 minutes at 4°C and resuspended in 300 μL cold nuclear lysis buffer (10 mM Tris-HCl at PH 8.0, 0.5 mM EGTA, 1 mM EDTA, 0.2% SDS), followed by rotation at 4°C for 20 minutes. Chromatin was sheared to produce 200–500 bp DNA fragments using a sonifier (Branson 150), with a setting of 15 pulses of 10 seconds each at 35% amplitude followed by a 40-second rest period on ice between each pulse. Samples were centrifuged at 16,000*g* for 10 minutes at 4°C to remove insoluble materials, and the resulting supernatant was subjected to a 2-fold dilution using nuclear lysis buffer. Diluted chromatin (5%–10%) samples were saved as input samples and stored at 4°C until use. To perform ChIP, 500 μL diluted chromatin was added to each tube containing antibody-bound beads and incubation on a rotator was carried out overnight at 4°C. Beads were washed 8 times with 1 mL cold wash buffer (100 mM Tris-HCl at pH 8.0, 500 mM NaCl, 1 % deoxycholic acid, 1% NP-40) by rotating at 4°C for 5 minutes each. Beads were eluted with 85 μL elution buffer (50 mM Tris-HCl at pH 8.0, 10 mM EDTA, 1% SDS) by agitation in a thermomixer at 65°C for 10 minutes. Elution was repeated once, and the 2 eluants were combined. The eluants and the input samples were incubated at 65°C for 4 hours to reverse the crosslinks. RNase A (10 μg) was added to each sample, and incubation was carried out for 1 hour at 37°C. Proteinase K (200 μg) dissolved in 120 μL TE buffer (50 mM Tris-HCl at pH 8.0, 10 mM EDTA) was added to each sample, and incubation was carried out for 2 hours at 65°C. Samples were purified using the QIAquick PCR Purification Kit (QIAGEN, 28104) and eluted in 30 μL ddH_2_O. ChIP-purified DNA levels were determined by qPCR. For ARC ChIP-qPCR, a previously reported primer set (93) was used. For SH-SY5Y cell ChIP-qPCR, a pair of house-designed primers was used. The sequences of both primer sets are listed in Supplemental Table 1. The relative enrichments of the DNA regions were calculated using the percentage input method and are presented as a percentage of input as previously described (48).

### IP experiments.

To prepare antibodies, 5 μL (packed volume) ChIP-grade Dynabeads Protein G (Invitrogen, Thermo Fisher Scientific, 10004D) were washed twice with 1 mL IP buffer (0.5% Triton X-100, 150 mM NaCl, 10 mM Tris–HCl at pH 7.5, and 10 mM EDTA), followed by incubation with 5 μg rabbit polyclonal anti-TET3 (Active Motif, 61395) (Supplemental Figure 6A) or preimmune rabbit IgG in 300 μL IP buffer at 4°C overnight. Antibody-bound beads were pelleted and kept on ice until use. To prepare lysate from ARCs (Figure 4F), PBS-washed ARCs (2 ARCs from 1 mouse per IP) freshly isolated from mice were homogenized (5–10 strokes) using a disposable pellet pestle in 500 μL freshly prepared gentle lysis buffer (GLB, 0.5% Triton X-100, 10 mM NaCl, 10 mM Tris–HCl at pH 7.5, 10 mM EDTA, and 1× protease inhibitor cocktail) and incubated on ice for 20 minutes with occasional inversion. To prepare lysate from GT1-7 (Figure 4G) and SH-SY5Y (Figure 4H), cells at a density of 6 × 10^6^ cells/well in a 100 mm plate were treated with Lept H (1 × 10^–8^ M) for 2 hours. Cells were then rinsed with cold PBS 3 times, collected by manual scraping in cold PBS, and pelleted by gentle centrifugation. The cell pellet was resuspended in 1 mL cold, freshly prepared GLB and incubated on ice for 20 minutes with occasional inversion. For IP of ARCs or cells, after centrifugation at 12,000*g* at 4°C for 15 minutes to remove insoluble materials, 5 M NaCl was added to a final concentration of 200 mM, and the lysate was transferred to a tube containing antibody/preimmune IgG–coated beads (400 μL lysate per IP). IP was carried out at 4°C for 4 hours. Following IP, the beads were quickly washed twice with 1 mL cold IP buffer and washed an additional 3 times with IP buffer by rotating at 4°C for 5 minutes each time. After the final wash, residual liquid was completely removed, and the beads were eluted with 16 μL 2× SDS buffer (containing 1× phosphatase inhibitor cocktail and 1× protease inhibitor cocktail) at 100°C for 5 minutes. Seven microliters per gel well of eluant was loaded onto a 4%–15% gradient SDS gel (Bio-Rad, 456-8086). For Western blot analysis, anti-TET3 (Genetex, GTX121453) (90), anti-STAT3 (Proteintech, 10253-2-AP) (91), anti–p-STAT3 (Cell Signaling Technology, 9145S Y705) (87), anti-NCOR1 (Cell Signaling Technology, 5948S) (94), and anti-HDAC4 (Active Motif, 40969) (Supplemental Figure 6B) antibodies were diluted at 1:1,000. The secondary antibody Rabbit IgG TrueBlot (1:1,000, Rockland, 18-8816-33) was used. These unique HRP-conjugated monoclonal secondary antibodies enabled the detection of immunoblotted target proteins without hindrance by interfering immunoprecipitating Ig heavy and light chains.

### Study approval.

All animal work was conducted in accordance with the guidelines of the Yale University IACUC, and the studies were approved by the IACUC of Yale University.

## Author contributions

YH conceived and supervised the project. DX, TLH, and YH designed the experiments, analyzed data, and wrote the manuscript. BS performed stereotaxic injections and mini-osmotic pump surgeries. FL, FC, and HL performed cell transfection and RNA and protein analyses. MSP performed behavioral tests. XBG and ZWL performed electrophysiology experiments and analyzed data. CS and GGC created the AAV-sgTet3 construct. JG and HZ performed bioinformatics analyses. JC and MS carried out indirect calorimetric analyses of animals. HST provided intellectual insights and critical discussion of the project.

## Supplementary Material

Supplemental data

## Version 1 10/03/2022

Electronic publication
