# Supplementary material for: TET3 epigenetically controls feeding and stress response behaviors via AGRP neurons
Source: J Clin Invest. 2022 Oct 3;132(19):e162365. doi: 10.1172/JCI162365 (PMC9525119; doi:10.1172/JCI162365)
Supplement: Supplemental data [file jci-132-162365-s008.pdf]

Supplemental Information for

TET3 epigenetically controls feeding and stress response behavior via AGRP neurons

Di Xie[1,2], Bernardo Stutz[2,3], Feng Li[1,2], Fan Chen[1], Haining Lv[1,2], Matija Sestan-Pesa[2,3], Jonatas Catarino[2,3], Jianlei Gu[4], Hongyu Zhao[4], Christopher Stoddard[5], Gordon G. Carmichael[5], Marya Shanabrough[2,3], Hugh S. Taylor[1], Zhong-Wu Liu[2,3], Xiao-Bing Gao[2,3], Tamas L. Horvath[1,2,3,6], Yingqun Huang[1,2]

Address correspondence to:

Yingqun Huang, Email: yingqun.huang@yale.edu; or Tamas Horvath, Email: tamas.horvath@yale.edu

This PDF file includes:

Supplemental Methods

Supplemental Figures 1-6

Supplemental Table 1

Supplemental Methods

Mouse

Both male and female mice were used for experiments. Mice were housed at 22°C-24°C with a 12 h light/12 h dark cycle with regular chow (Harlan Teklad no. 2018, 18% calories from fat) and water provided ad libitum. C57BL/6J (Jax, 000664), *Agrp-IRES-Cre* (1) (Jax, 012899), and *Rosa26-LSL-Cas9-GFP* (2) (Jax, 026175) were purchased from the Jackson Laboratory. Following stereotaxic injection to express AAVs, mice were individually housed with ad libitum access to regular chow and water. Littermates of the same sex were randomly assigned to either control or experimental groups. For all experiments, age- and sex-matched animals were used. For information on animal numbers, refer to figure legends.

Viruses

The AAV-sgTet3 (pAAV-sgRNA-Tet3-pEF1a-DIO mCherry) was constructed based on pAAV-pEF1α-DIO EYFP plasmid, a gift from Karl Deisseroth (Addgene #27056). NHE I and BSRG I (New England Biolabs) were used to replace the EYFP with an mCherry fragment using the same sites. This vector was then linearized with MLU I for a later ligation. The guide RNA was made by phosphorylating and annealing overlapping oligos from Integrated DNA Technologies and cloned into the bbs1 digested pSpCas9(BB)-2A-Puro (PX459V2) plasmid, a gift from Feng Zhang (Addgene # 48139) (3). The U6 promoter, guide RNA and gRNA scaffold were PCR amplified and cloned into the MLU I site in the above digested vector. The resulting AAV-sgTet3 was packaged at Vigene Biosciences, Inc. The pAAV-hSyn-DIO-mCherry (AAV) (Addgene viral prep # 50459-AAV9) and pAAV-hSyn-DIO-hM4D(Gi)-mCherry (AAV-h4MDi) (4) (Addgne viral prep #44362-AAV9) were gifts from Bryan Roth. Viral particles were resuspended in calcium/magnesium-free DPBS (Gibco, catalog # 14190144) at $2x10^{13}$ GC/ml and viral aliquots were stored at −80 ^{0}C before stereotaxic injection. The viruses were freshly diluted using DPBS before stereotaxic injection.

Leptin treatment of mice

To assess leptin effects on suppression of hunger-induced appetite, female Cas9+ mice injected with AAV or AAV-sgTet3 bilaterally into the ARC were fasted overnight for 22 h. On the second day, mouse leptin (L3772-1MG, Sigma Aldrich) was administrated at 5 mg/kg intraperitoneally at 10:00 and pre-weighed food was placed

in the cage and monitored for the following 24 h. For ChIP studies, Cas9+ mice injected with AAV or AAV-sgTet3 bilaterally into the ARC were fasted overnight for 22 h. On the second day, saline or leptin (5 mg/kg) was administrated intraperitoneally at 10:00. Two hours later, ARCs were isolated for ChIP-qPCR analysis. To examine leptin-induced protein-protein interactions, Cas9+ mice were fasted overnight for 22 h. On the second day, leptin was administrated at 5 mg/kg intraperitoneally at 10:00, and ARCs were isolated 2 h later, followed by co-IP studies.

Stereotaxic injection

Injections were made into the ARC of anesthetized 6-week-old Cas9+ mice, placed in a stereotaxic apparatus (model 902; Kopf Instruments). Viruses (500 nL, 5×10^{12} GC/ml per site of injection) were applied into each hemisphere (coordinates: bregma, anterior-posterior: −1.45 mm, dorsal-ventral: −5.8 mm, lateral: +/- 0.27 mm) by using an air pressure system (injection time: 5 minutes). After surgery, mice were allowed to recover for 2 weeks before electrophysiological recording. Stereotaxic injection sites were verified by double fluorescence labeling for GFP and mCherry, which could be detected without immunostaining. Mice with “missed” or “partial” hits were excluded from data analyses.

Osmotic pump installation

Three days after bilateral ARC co-injection with AAV-sgTet3 and AAV-h4MDi, a mini-osmotic pump (model 1007D, Alzet) was implanted subcutaneously. The osmotic pump was filled with either sterile saline solution or DREADD agonist compound 21 (C21) dihydrochloride (0.5 mg/kg, HB6124-25mg, Hello Bio). Food intake measurement and ITT were performed at day 5 and day 9 postinjection, respectively. For food intake assays, food pellets were weighed at 10:00 each day for 3 continuous days and an average of three-day food intake was calculated.

Body weight, body composition, and food intake measurement

Mice were singly housed after surgery. Body weight was measured every other week, and body composition was assessed using EchoMRI analysis. Food intake and energy expenditure were measured using an indirect calorimetry chamber (TSE Systems, Germany).

Electrophysiology

Coronal hypothalamic slices containing the ARC were prepared from virus injected mice as previously reported (5). In brief, mice were anesthetized with isoflurane and decapitated. The brain was rapidly removed and immersed in cold (4 ºC) and oxygenated cutting solution containing (in mM): sucrose 220, KCl 2.5, NaH_2PO_4 1.23, $NaHCO_3$ 26, $CaCl_2$ 1, $MgCl_2$ 6, and glucose 10 (pH 7.3 with NaOH). Coronal slices (300 µm thick) were prepared with a Leica vibratome after the brain was trimmed to a small tissue block containing the hypothalamus. After preparation, slices were maintained at room temperature (23 ºC –25 ºC) in a storage chamber in artificial cerebrospinal fluid (ACSF) (bubbled with 5% CO_2 and 95% O_2) containing (in mM): NaCl 124, KCl 3, $CaCl_2$ 2, $MgCl_2$ 2, NaH_2PO_4 1.23, $NaHCO_3$ 26, glucose 10 (pH 7.4 with NaOH) for recovery and storage. After recovery at room temperature for at least 1 hour, slices were transferred to a recording chamber constantly perfused at a rate of 2 mL/min with ACSF containing 2.5 mM glucose at a temperature of 33 ºC for electrophysiological experiments. To identify virus infected AGRP neurons, mCherry and GFP fluorescence were detected using LED illumination (CoolLED pE-300). Whole-cell patch clamp recordings were obtained from AGRP neurons visualized using infrared differential interference contrast (IR-DIC) imaging. Spontaneous membrane and action potentials (MP) were recorded under current clamp as previously reported (6, 7). The micropipettes (4–6 MΩ) were made of borosilicate glass (World Precision Instruments) with a micropipette puller (Sutter P-97) and backfilled with a pipette solution containing (in mM): K-gluconate 108, KCl 27, $MgCl_2$ 2, HEPES 10, EGTA 1.1, Mg-ATP 2.5, Na_2-GTP 0.3, and Na_2-phosphocreatine 10, pH 7.3 with KOH. Both input resistance and series resistance were monitored throughout the experiments, and the former was partially compensated. Only recordings with stable series resistance and input resistance were accepted. All data were sampled at 3 kHz, filtered at 3 kHz, and analyzed with an Apple Macintosh computer using AxoGraph X. t test was used to examine the statistical significance of the difference in AP frequency and threshold in the recorded AGRP neurons.

Bobcat339 treatment of mice

Chow-fed C57BL/6J mice at the age of 12 weeks were treated with Bobcat339 (100 mg/kg per day) or vehicle (DMSO) in drinking water for 4 days. ARCs were isolated at 10:00 from ad libitum-fed mice and subjected to

immunofluorescence analysis. Bobcat339 was dissolved in DMSO at a concentration of 100 mg/ml and stored at -20 ^{0}C in aliquots. Working solution (1 mg/ml) was freshly prepared every other day by dilution using tap water.

GTT and ITT

Glucose tolerance tests (GTT) were performed following 16 h overnight fasting. Each animal received an intraperitoneal injection of 2 g/kg glucose (Sigma-Aldrich, G5767) in sterile saline. Insulin tolerance tests (ITT) were performed following a 3 h morning-fasting. Each animal received an intraperitoneal injection of 1 U/kg insulin (Novolin R Regular U-100 insulin) in sterile saline. Blood glucose concentrations were measured using Contour next blood glucose meter (Ascensia Diabetes Care) via tail vein bleeding at the indicated time points after injection.

Western blot analysis

GT1-7 and SH-SY5Y cells in 24-well plates ($2.5x10^5$ cells/well) were rinsed with cold PBS three times and collected by manual scraping in 150 μl of 2x SDS-sample buffer containing 1X Phosphatase inhibitor cocktail (Thermo, 78427) and 1X Protease inhibitor cocktail (Thermo, 78438), followed by heating at 100°C for 5 min with occasional vortexing. The lysate was then centrifuged at 12,000 g for 5 min to remove insoluble materials before loading onto a 4-15% gradient SDS gel (Bio-rad, 456-8086) (10 μl/well), followed by Western blot analysis. The antibodies used were anti-TET3 (diluted at 1:1000; mouse/human, GeneTex, GTX121453) (8) (Supplemental Figure 6A), anti-AGRP (mouse) (diluted at 1:500; MilliporeSigma, AB3402P) (validated by the vendor), anti-AGRP (human) (diluted at 1:500; Abcam, Ab113481) (9, 10), anti-NPY (diluted at 1:1000; mouse/human, Cell Signaling 11976S) (11), anti-VGAT (diluted at 1:4000; mouse/human, Abcam, Ab235952) (validated by the vendor), and HRP-conjugated anti-GAPDH (diluted at 1:5000; Proteintech, HRP-60004). The secondary antibody was HRP-linked Anti-rabbit IgG (Cell Signaling, 7074).

Hydroxymethylated DNA immunoprecipitation coupled with qPCR (hMeDIP-qPCR)

The experiments were carried out using the EpiQuik hMeDIP Kit (P-1038-48, Epigentek) according to the manufacturer's instructions. Briefly, for ARC hMeDIP, freshly isolated ARCs (2 ARCs from one mouse per IP,

Figure 4K) were washed twice with 1 ml of cold PBS and homogenized (5-10 strokes) using a disposable pellet pestle (Fisher Scientific, 12-141-368) in 500 µl of Genomic Lysis Buffer. For SH-SY5Y hMeDIP (Figure 4L), cells seeded in 6-well plates at 1×10^6 cells/well the night before were transfected with NT siRNA or *TET3* siRNA under Lept H conditions, and genomic DNAs were isolated at 48 h following transfection using Quick gDNA MicroPrep Kit (D3021, Zymo Research Corporation) and sheared using a sonifier (Branson 150), with a setting of 9 pulses of 10 sec each at 35% amplitude followed by a 40 sec rest period on ice between each pulse. Sheared DNA fragments (ranged in size from 200-600 bps as assessed by agarose gel electrophoresis) were immunoprecipitated using the 5hmC rabbit polyclonal antibody from the kit. qPCR was performed in a 25 μl reaction containing 2.5 μl of the eluted DNA using iTAC SYBGreen in a Bio-Rad iCycler. The relative enrichments (after normalization against control IgG) of the indicated DNA regions were calculated using the Percent Input Method according to the manufacturer's instructions.

Plasma insulin, leptin, and corticosterone

For insulin and leptin, blood samples were collected in EDTA tubes (Microtainer with K2EDTA, BD, 365974) by cardiac puncture of terminally anesthetized animals between 9:00 and 11:00. For corticosterone, blood samples were obtained via retroorbital bleeding between 19:00 and 20:00. The tubes were centrifuged at 2,000 x g at 4 °C for 20 min, and plasma was collected and stored at -80 °C until use. Plasma insulin, leptin, and corticosterone levels were measured using Mouse Insulin ELISA kit (Crystal Chem, 90080), Mouse Leptin ELISA kit (Crystal Chem,90030), and Corticosterone ELISA kit (Enzo, ADI-900-097), respectively, according to the manufacturer's instructions.

Behavioral Tests

For all behavioral tests, mice were transferred to the testing room 1 h prior to testing for acclimation to the environment. All behavioral apparatus was wiped with 70% ethanol prior to each trial and between trials. The tail suspension test (TST) (12) and the forced swim test (FST) (13) lasted for 6 min and the total amount of immobility time was measured for each animal and considered as an index of "depressive-like" behavior. For the TST, cylindrical plastic tubes were placed at the base of the tail to prevent tail climbing.

Statistical Analysis

All statistical analyses were performed using GraphPad Prism version 8 for Windows (GraphPad Software, La Jolla California USA, www.graphpad.com) and are presented as mean ± SEM. Two-tailed Student's t tests (or as otherwise indicated) were used to compare means between groups. $P < 0.05$ was considered significant.

References

1. Tong Q, Ye CP, Jones JE, Elmquist JK, and Lowell BB. Synaptic release of GABA by AgRP neurons is required for normal regulation of energy balance. *Nat Neurosci.* 2008;11(9):998-1000.
2. Platt RJ, Chen S, Zhou Y, Yim MJ, Swiech L, Kempton HR, et al. CRISPR-Cas9 knockin mice for genome editing and cancer modeling. *Cell.* 2014;159(2):440-55.
3. Ran FA, Hsu PD, Wright J, Agarwala V, Scott DA, and Zhang F. Genome engineering using the CRISPR-Cas9 system. *Nat Protoc.* 2013;8(11):2281-308.
4. Krashes MJ, Koda S, Ye C, Rogan SC, Adams AC, Cusher DS, et al. Rapid, reversible activation of AgRP neurons drives feeding behavior in mice. *J Clin Invest.* 2011;121(4):1424-8.
5. Varela L, Stutz B, Song JE, Kim JG, Liu ZW, Gao XB, et al. Hunger-promoting AgRP neurons trigger an astrocyte-mediated feed-forward autoactivation loop in mice. *J Clin Invest.* 2021;131(10).
6. Tan Y, Hang F, Liu ZW, Stoiljkovic M, Wu M, Tu Y, et al. Impaired hypocretin/orexin system alters responses to salient stimuli in obese male mice. *J Clin Invest.* 2020;130(9):4985-98.
7. Liu ZW, Gan G, Suyama S, and Gao XB. Intracellular energy status regulates activity in hypocretin/orexin neurones: a link between energy and behavioural states. *J Physiol.* 2011;589(17):4157-66.
8. Xu Y, Sun X, Zhang R, Cao T, Cai SY, Boyer JL, et al. A Positive Feedback Loop of TET3 and TGF-beta1 Promotes Liver Fibrosis. *Cell Rep.* 2020;30(5):1310-8 e5.
9. Lopez R, Arumugam A, Joseph R, Monga K, Boopalan T, Agullo P, et al. Hyperglycemia enhances the proliferation of non-tumorigenic and malignant mammary epithelial cells through increased leptin/IGF1R signaling and activation of AKT/mTOR. *PLoS One.* 2013;8(11):e79708.
10. Imbernon M, Sanchez-Rebordelo E, Gallego R, Gandara M, Lear P, Lopez M, et al. Hypothalamic KLF4 mediates leptin's effects on food intake via AgRP. *Mol Metab.* 2014;3(4):441-51.
11. Glaser J, Iranzo J, Borensztein M, Marinucci M, Gualtieri A, Jouhanneau C, et al. The imprinted Zdbf2 gene finely tunes control of feeding and growth in neonates. *Elife.* 2022;11.
12. Steru L, Chermat R, Thierry B, and Simon P. The tail suspension test: a new method for screening antidepressants in mice. *Psychopharmacology (Berl).* 1985;85(3):367-70.
13. Yankelevitch-Yahav R, Franko M, Huly A, and Doron R. The forced swim test as a model of depressive-like behavior. *J Vis Exp.* 2015(97).

Supplemental Figures

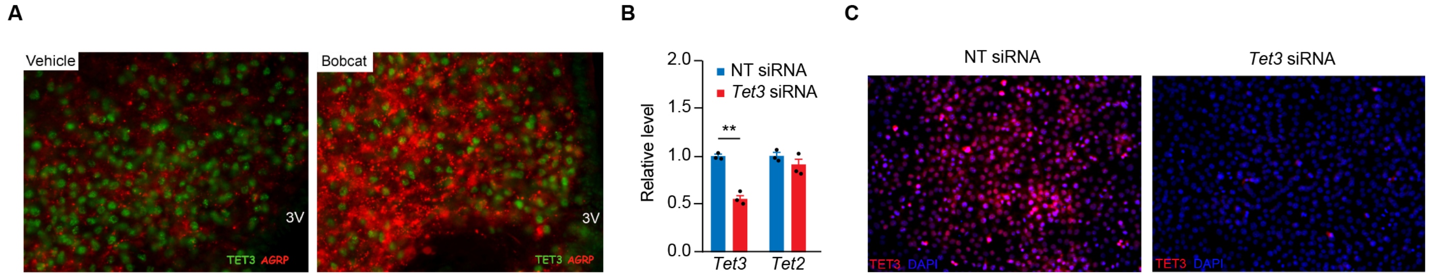

Supplemental Figure 1. Bobcat treatment and siRNA-mediated TET3 knockdown. (**A**) Representative photomicrographs showing increased AGRP (red) in the ARC of mice treated with Bobcat vs. vehicle. TET3 (green) is a predominantly nuclear protein. As AGRP neurons were not labeled, the TET3-positive cells represented both AGRP and non-AGRP cells. 3V, third ventricle. (**B**) Levels of *Tet3* and *Tet2* mRNAs in GT1-7, a mouse hypothalamic cell line, transfected with non-targeting control siRNA (NT siRNA) or siRNA specifically targeting mouse *Tet3* (*Tet3* siRNA) for 48 h. *n* = 3 per group. Data: mean ± SEM. **p < 0.01, by 2-tailed Student's t tests. (**C**) Representative photomicrographs showing reduced TET3 protein (red) in GT1-7 cells treated with *Tet3* siRNA vs. NT siRNA for 48 h. Cell nuclei (blue) were labeled by DAPI.

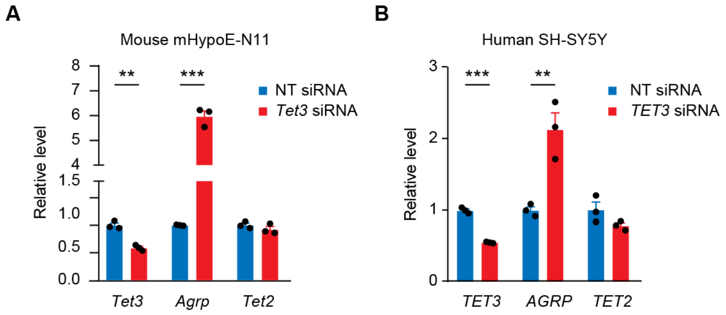

Supplemental Figure 2. TET3 negatively regulates AGRP expression both in mouse and human cell lines. Data: mean ± SEM. (**A**) Levels of *Tet3*, *Agrp* and *Tet2* mRNAs in mouse mHypoE-N11 embryonic hypothalamus cell line transfected with NT siRNA or *Tet3* siRNA. RNAs were extracted at 24 h (for *Tet3* and *Tet2*) or at 48 h (for *Agrp*). *n* = 3 per group. **p < 0.01, ***p < 0.001, by 2-tailed Student's t tests. (**B**) Levels of *TET3*, *AGRP* and *TET2* mRNAs in human SH-SY5Y neuroblastoma cell line transfected with NT siRNA or siRNA specifically targeting human *TET3* (TET3 siRNA). RNAs were extracted at 24 h (for *TET3* and *TET2*) or at 48 h (for *AGRP*). *n* = 3 per group. **p < 0.01, ***p < 0.001, by 2-tailed Student's t tests.

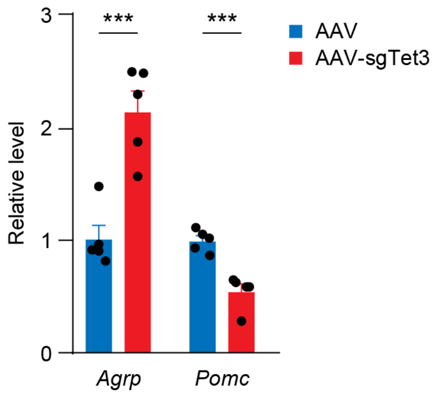

Supplemental Figure 3. Levels of *Agrp* and *Pomc* mRNAs in the ARCs of ad libitum-fed Cas9+ mice injected with AAV or AAV-sgTet3. *n* = 5 mice per group. Data: mean ± SEM. ****P* < 0.001, by 2-tailed Student's t tests.

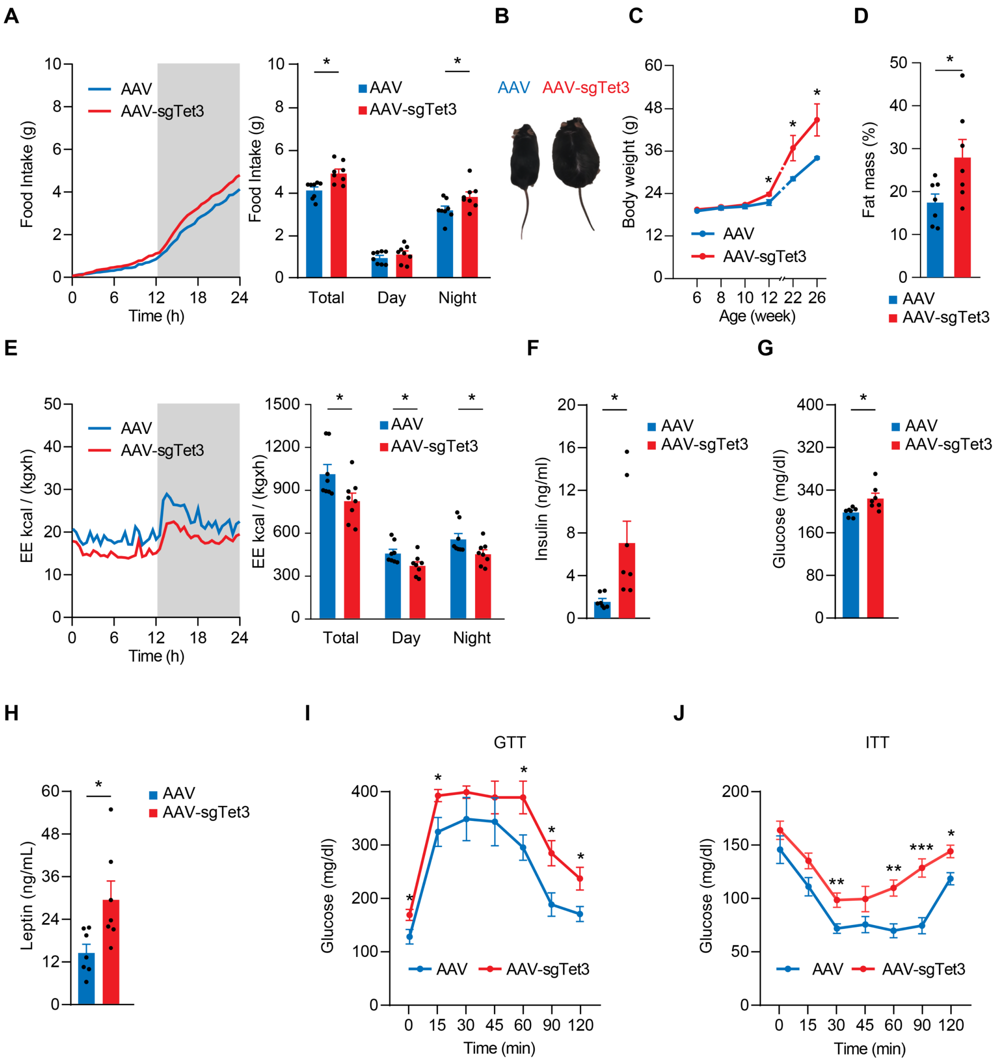

Supplemental Figure 4. **AgRP neuron-specific TET3 knockdown in male mice induced hyperphagia, obesity and diabetes.** Data: mean ± SEM. (**A**) Cas9+ mice injected with AAV-sgTet3 or AAV bilaterally into the ARC at the age of 6 weeks became hyperphagic at 3 weeks post injection. *n* = 8 animals per group. **P* < 0.05, by 2-tailed Student's t tests. (**B**) Representative images of mice at 8 weeks post injection. (**C**) Body weight changes of mice post injection. *n* = 8 animals per group. **P* < 0.05, by 2-tailed Student's t tests. (**D**) Fat mass of mice at 8 weeks post injection. *n* = 8 animals per group. **P* < 0.05, by 2-tailed Student's t tests. (**E**) Energy expenditure at 3 weeks post injection. *n* = 8 animals per group. **P* < 0.05, by 2-tailed Student's t tests. (**F**) Ad libitum-fed blood insulin at 6 weeks post-injection. *n* = 8 animals per group. **P* < 0.05, by 2-tailed Student's t tests. (**G**) Ad libitum-fed blood glucose at 7 weeks post injection. *n* = 8 animals per group. **P* < 0.05, by 2-tailed Student's t tests. (**H**) Ad libitum-fed blood leptin at 7 weeks post-injection. *n* = 8 animals per group. **P* < 0.05, by 2-tailed Student's t tests. (**I**) Glucose tolerance tests (GTT) at 8 weeks post injection. *n* = 8 animals per group. **P* < 0.05, by 2-way ANOVA with Sidak post-test. (**J**) Insulin tolerance tests (ITT) at 9 weeks post-injection. *n* = 8 animals per group. **P* < 0.05, ***P* < 0.01, by 2-way ANOVA with Sidak post-test.

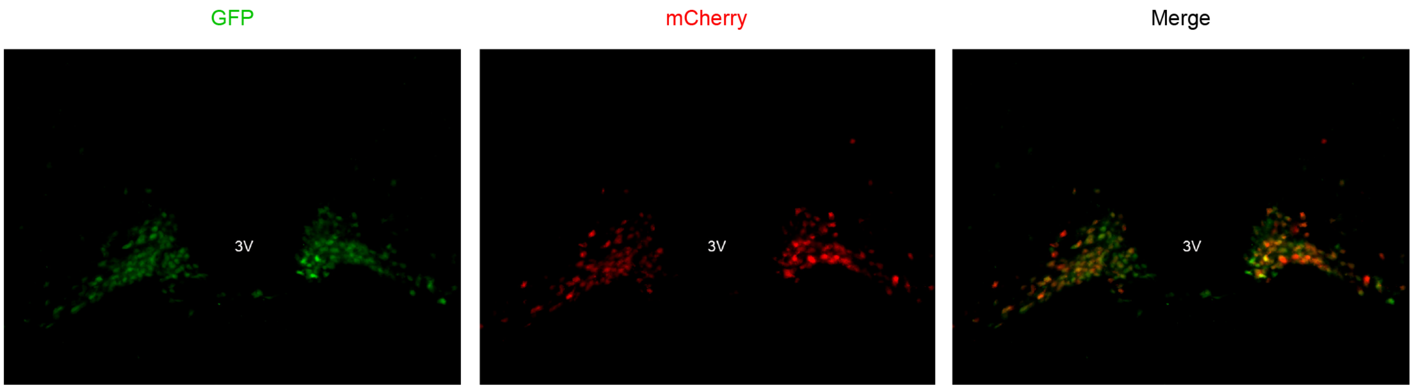

Supplemental Figure 5. **AgRP neuron-specific expression of hM4Di.** AAV-hM4Di was injected bilaterally into the ARC of Cas9+ mice, followed by detection of AgRP neurons (green) expressing AAV-hM4Di (red) without immunostaining.

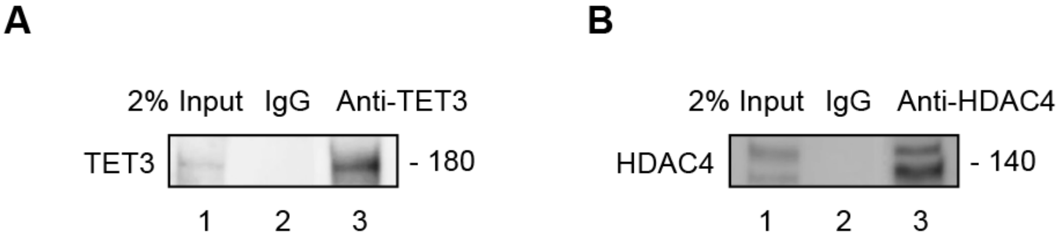

Supplemental Figure 6. **Additional validation of antibodies specific for TET3 and HDAC4 in immunoprecipitation and western blot analyses.** (**A**) Immunoprecipitation experiments were performed with mouse GT1-7 cells using Anti-TET3 antibody (Active Motif, 61395) or pre-immune IgG (as a negative control) showing that the Anti-TET3 was able to pull down endogenous TET3 which could be detected by a different TET3 antibody (GeneTex, GTX121453) in western blot analysis. (**B**) Immunoprecipitation experiments were performed with mouse GT1-7 cells using Anti-HDAC4 antibody (Active Motif, 40969) showing that this antibody was able to pull down endogenous HDAC4 which could be detected by the same HDAC antibody in western blot analysis.

Supplemental Table 1

qPCR primer sequences		
**Gene**	**Forward Primer**	**Reverse Primer**
Rplp0 (mouse)	5'-GATGGGCAACTGTACCTGACTG-3'	5'-CTGGGCTCCTCTTGGAATG-3'
Agrp (mouse)	5'-GGCCTCAAGAAGACAACTGC-3'	5'-GCAAAAGGCATTGAAGAAGC-3'
Tet3 (mouse)	5'-GCCGATGCAGTAGTGGAGG-3'	5'-CTGCCTTGAATCTCCATGGTAC-3'
Tet2 (mouse)	5'-AGCAAGAGATTCCGAAGGAT-3'	5'-AGTGGAGGACTGAGTGCAAG-3'
Npy(mouse)	5'-AGGCTTGAAGACCCTTCCAT-3'	5'-ACAGGCAGACTGGTTTCAGG-3'
Pomc (mouse)	5'-CATCTTTGTCCCCAGAGAGC-3'	5'-GCACCAGCTCCACACATCTA-3'
Slc32a1(mouse)	5'-TGGTCATCGCTTACTGTCTC-3'	5'-TGCTGCATGTTGCCTTCG-3'
RPLP0 (human)	5'-GGCGACCTGGAAGTCCAACT-3'	5'-CCATCAGCACCACAGCCTTC-3'
AGRP (human)	5'-GAAGAGGATCTGTTGCAGGA-3'	5'-CAGGACTCATGCAGCCTTAC-3'
TET3 (human)	5'-GACGAGAACATCGGCGGCGT-3'	5'-GTGGCAGCGGTTGGGCTTCT-3'
TET2 (human)	5'-TTCGCAGAAGCAGCAGTGAAGAG-3'	5'-AGCCAGAGACAGCGGGATTCCTT-3'
NPY (human)	5'-TCACCAGGCAGAGATATGGA-3'	5'-GCAAGTCTCATTTCCCATCA-3'
SLC32A1(human)	Bio-Rad, qHsaCED0042869	
**ChIP-qPCR / hMeDIP-qPCR primer sequences**		
**Gene**	**Forward Primer**	**Reverse Primer**
Agrp(mouse)	5'-GTGCCCTTGACAAAGTTCCTGGAA-3'	5'-GCAGAACCTAGGGATGGGTCATGC-3'
AGRP(human)	5'-GAATTCTTGGAAGCACAGGAAACA-3'	5'-CAGGGACCTAGGAGCTGAGCAGGG-3'
